# Clinical validation of p16/Ki‐67 dual‐stained cytology triage of HPV‐positive women: Results from the IMPACT trial

**DOI:** 10.1002/ijc.33812

**Published:** 2021-09-25

**Authors:** Thomas C. Wright, Mark H. Stoler, Jim Ranger‐Moore, Qijun Fang, Patrick Volkir, Mahboobeh Safaeian, Ruediger Ridder

**Affiliations:** ^1^ Department of Pathology and Cell Biology Columbia University New York New York USA; ^2^ Department of Pathology University of Virginia Health System Charlottesville Virginia USA; ^3^ Ventana Medical Systems, Inc/Roche Tissue Diagnostics Tucson Arizona USA; ^4^ Roche Molecular Solutions, Inc Pleasanton California USA; ^5^ Roche Molecular Systems, Inc Pleasanton California USA

**Keywords:** cervical cancer, HPV testing, p16/Ki‐67 dual‐stain

## Abstract

Triage strategies are needed for primary human papillomavirus (HPV)‐based cervical cancer screening to identify women requiring colposcopy/biopsy. We assessed the performance of p16/Ki‐67 dual‐stained (DS) immunocytochemistry to triage HPV‐positive women and compared it to cytology, with or without HPV16/18 genotyping. A prospective observational screening study enrolled 35 263 women aged 25 to 65 years at 32 U.S. sites. Cervical samples had HPV and cytology testing, with colposcopy/biopsy for women with positive tests. Women without cervical intraepithelial neoplasia Grade 2 or worse (≥CIN2) at baseline (n = 3876) were retested after 1 year. In all, 4927 HPV‐positive women with valid DS results were included in this analysis. DS sensitivity for ≥CIN2 and ≥CIN3 at baseline was 91.2% (95% confidence interval [CI]: 86.8%‐94.2%) and 91.9% (95% CI: 86.1%‐95.4%), respectively, in HPV16/18‐positive women and 83.0% (95% CI: 78.4%‐86.8%) and 86.0% (95% CI: 77.5%‐91.6%) in women with 12 “other” genotypes. Using DS alone to triage HPV‐positive women showed significantly higher sensitivity and specificity than HPV16/18 genotyping with cytology triage of 12 “other” genotypes, and substantially higher sensitivity but lower specificity than using cytology alone. The risk of ≥CIN2 was significantly lower in HPV‐positive, DS‐negative women (3.6%; 95% CI: 2.9%‐4.4%), compared to triage‐negative women using HPV16/18 genotyping with cytology for 12 “other” genotypes (7.4%; 95% CI: 6.4%‐8.5%; *P* < .0001) or cytology alone (7.5%; 95% CI: 6.7%‐8.4%; *P* < .0001). DS showed better risk stratification than cytology‐based strategies and provided high reassurance against pre‐cancers both at baseline and at 1‐year follow‐up, irrespective of the HPV genotype. DS allows for the safe triage of primary screening HPV‐positive women.

## Abstract 2

What's new?

Primary screening for human papillomavirus (HPV) requires efficient triage of HPV‐positive women to colposcopy and biopsy. In this prospective observational trial in the United States, with 1‐year longitudinal follow‐up, the authors investigated the performance of p16/Ki‐67 dual‐stain cytology for the triage of women identified as HPV‐positive during primary screening. Compared to HPV16/18 genotyping combined with cytological triage of other HPV genotypes, dual‐stain cytology was significantly more sensitive for predicting risk of cervical intraepithelial neoplasia grade 2/3 or worse. The findings indicate that dual‐stain cytology is effective for triage of HPV‐positive women, either alone or when combined with partial HPV genotyping.

**Abbreviations**
ACIS: adenocarcinoma in situ
AGC: atypical glandular cells
ASCCP: American Society for Colposcopy and Cervical Pathology
ASC‐H: atypical squamous cells—cannot exclude HSIL
ASC‐US: atypical squamous cells of undetermined significance
CI: confidence interval
CIN: cervical intraepithelial neoplasia
CPR: central pathology review
DS: dual stain
H&E: hematoxylin and eosin
HPV: human papillomavirus
HSIL: high‐grade squamous intraepithelial lesion
KPNC: Kaiser Permanente Northern California
LSIL: low‐grade squamous intraepithelial lesion
NILM: negative for intraepithelial lesion or malignancy
NPV: negative predictive value
PPV: positive predictive value
SD: standard deviation

## 1 INTRODUCTION

Molecular testing for human papillomavirus (HPV) is now widely accepted as the preferred approach for cervical cancer screening. A number of countries including Australia, United Kingdom, the Netherlands, Sweden, Denmark and Turkey have phased out Pap cytology (cytology) as the primary cervical cancer screening test and replaced it with primary HPV testing. The biggest challenge to implementing primary HPV screening is managing the large number of women found to have transient HPV infections. In large U.S. cervical cancer screening trials, approximately 14% of women 25 years and older are HPV positive.^1^, ^2^, ^3^ Efficient triage methods are needed to determine which HPV‐positive women are at increased risk of high‐grade cervical cancer precursors or cancer and require colposcopy as opposed to those who need follow‐up with repeat testing or routine screening.^4^ Cervical cytology has been used to triage HPV‐positive women but because of its low sensitivity for high‐grade precursors, cytology‐negative women need to be retested at a short interval.^5^ HPV16/18 genotyping is also used in some settings for triage due to the elevated risk of high‐grade precursors and invasive cancers associated with these genotypes. Triage with HPV16/18 genotyping alone also has limited sensitivity since only approximately 50% of high‐grade cervical cancer precursors are associated with these genotypes.^6^ To address this limitation, HPV16/18 genotyping has been combined with cytological triage of women with the 12 “other” HPV genotypes. However, the limited sensitivity of cytology means that a relevant proportion of women with the 12 “other” genotypes with a negative cytology may have precancer.

Testing for the presence of cervical cells showing simultaneous expression of both the cell‐cycle regulator protein p16 and the proliferation‐associated Ki‐67 protein (p16/Ki‐67 dual‐stained cytology, ie, DS) has been shown in multiple studies to provide good specificity while maintaining high sensitivity when used as a triage test for abnormal cytology or positive HPV screening test results.^7^, ^8^, ^9^, ^10^, ^11^, ^12^, ^13^, ^14^, ^15^, ^16^, ^17^ This manuscript provides the results from the IMproved Primary screening And Colposcopy Triage (IMPACT) trial for the clinical performance of DS for the triage of HPV‐positive women in a large primary HPV screening population in the United States. The clinical performance of DS is compared to triage using HPV16/18 genotyping combined with cervical cytology or cytology alone.

## 2 MATERIALS AND METHODS

### 2.1 Patient enrolment

Women aged 25 to 65 years attending routine cervical cancer screening visits at 32 clinical sites offering cervical cancer screening services, including Planned Parenthood clinics, in 16 states across the United States between September 2017 and November 2018 were invited to join the IMPACT trial, as previously described in detail.^3^ Subjects willing and able to provide written informed consent were eligible unless they were pregnant, had a known history of ablative or excisional cervical therapy within the past 12 months, known history of hysterectomy or current or planned participation in another cervical cancer screening, treatment or vaccination study. Women were referred to colposcopy and biopsy/endocervical curettage within 12 weeks after enrolment if test results showed abnormal cytology (ie, ASC‐US or worse), a positive HPV test result or combined unsatisfactory cytology and HPV‐negative test results. All study‐related costs including costs for cytology and HPV testing, costs for colposcopy visits and biopsy evaluations, as well as costs for treatment performed according to the study protocol were covered by the sponsor of the trial (Roche).

The IMPACT trial consisted of two phases, a baseline (cross‐sectional) and a 1‐year follow‐up phase. Women who met the clinical endpoint (ie, biopsy‐confirmed ≥CIN2 [cervical intraepithelial neoplasia Grade 2] after the baseline colposcopy/biopsy visit) exited the study. Women who did not meet the primary endpoint and/or did not undergo treatment at baseline were invited to participate in the follow‐up phase of the trial. Subjects included in the follow‐up phase underwent an additional round of HPV and cytology testing after 12 months and, analogous to baseline procedures, were referred to colposcopy/biopsy if positive for either of these tests. The flow of the subjects through the baseline and 1‐year follow‐up phases of the IMPACT trial is shown in Figure S1.

### 2.2 Test methods

Women had one cervical sample collected into a liquid‐based cytology vial (PreservCyt; Hologic Inc, Marlborough, MA) using either spatula/brush or broom‐type collection devices (approximately half of the cohort per device). Specimens were shipped to 1 of 4 central laboratories in the United States participating as clinical laboratory study sites for the trial, and all laboratory testing was performed by these four laboratories.

HPV testing using both the cobas 4800 HPV Test and cobas HPV for use on the cobas 6800/8800 Systems (cobas 6800/8800 HPV test; Roche Molecular Systems, Inc, Pleasanton, CA) and cytology testing using the ThinPrep Pap Test (Hologic, Inc) were performed on all women enrolled into the IMPACT trial, according to the respective manufacturer's instructions. The use of both cobas 4800 and 6800/8800 HPV tests (each of them providing separate results for HPV16, HPV18 and the 12 “other” HPV types as a group) on every women allowed for the assessment of the performance of the high‐throughput cobas 6800/8800 HPV test compared to the cobas 4800 HPV test in primary HPV screening, co‐testing with cytology and ASC‐US triage, as described in more detail recently.^3^ Furthermore, it allowed us to establish the performance characteristics of DS in triaging women tested positive using either cobas HPV test. Residual cell suspension material in the PreservCyt vials from all women who were referred to colposcopy/biopsy at baseline were tested for the presence of p16/Ki‐67 dual‐stained cervical cells using the CINtec *PLUS* Cytology kit (Ventana Medical Systems, Inc, Tucson, AZ) on BenchMark ULTRA automated instruments according to the manufacturer's instructions. For the interpretation of p16/Ki‐67 DS slides, at least two cytotechnologists and at least two pathologists from each of the four clinical laboratory sites participated in the review of the slides. Every p16/Ki‐67 DS cytology slide was first interpreted by one cytotechnologist, and the final test result was confirmed by one pathologist.

### 2.3 Study cohorts

For the assessment of the performance of DS in triaging HPV‐positive women, only women with positive HPV test results at baseline were included in the analyses. Results for the analysis of DS and comparators as triage tests for women positive for the cobas 6800/8800 HPV test are reported in the main body of the manuscript, whereas results for the cohort of cobas 4800 HPV positive women are tabulated in the Supplementary Appendix.

### 2.4 Clinical endpoints

Clinical endpoints for the study were biopsy‐confirmed ≥CIN2 (ie, CIN2, CIN3, adenocarcinoma in situ [ACIS] and cervical cancer; primary endpoint) and ≥CIN3 (secondary endpoint). Formalin‐fixed, paraffin‐embedded biopsy tissue specimens were used for preparation of hematoxylin and eosin (H&E)‐stained slides, as well as for p16 immunohistochemical staining using the CINtec Histology kit (Ventana Medical Systems, Inc) according to the manufacturer's instructions. The pathology review result of the respective clinical laboratory was used for clinical management of the patients. For study purposes, all tissue specimens were subjected to a central pathology review (CPR) as previously described in detail.^3^ CPR results on H&E with p16‐stained slides added to the review per Lower Anogenital Squamous Terminology (LAST) criteria (but without using HPV16/18‐positive ASC‐US as an inclusion criterion) were used as the primary reference diagnoses for the trial.^18^

### 2.5 Study objectives and statistical methods

Co‐primary objectives for the IMPACT trial were (a) to evaluate the performance of DS for identification of ≥CIN2/≥CIN3 when used to triage HPV‐positive women, stratified by HPV16/18 vs 12 “other” HPV genotypes, and (b) to compare the performance of DS to that of cytology when used to triage 12 “other” HPV‐positive women. Acceptable performance of DS for the first objective required 1‐negative predictive value (NPV) for ≥CIN3 (ie, the risk of ≥CIN3 among DS‐negative women) to be ≤5% for HPV16/18‐positive women. Acceptable performance for the second objective required the same for 12 “other” HPV‐positive women or, if not met, then 1‐NPV for DS be no worse than cytology for the triage of 12 “other” HPV‐positive women.

Statistical analyses were performed on the intended use population of HPV‐positive women included in the IMPACT trial using SAS software, version 9.4.^19^ CPR results were tabulated by joint distribution of cytology (negative for intraepithelial lesion or malignancy [NILM], ASC‐US, AGC/ASC‐H, low‐grade squamous intraepithelial lesion [LSIL], HSIL/ACIS), HPV (HPV16+, HPV18+, HPV16/18+, 12 other HR‐HPV positive) and DS (DS+, DS−) results. Sensitivity, specificity, positive predictive value (PPV) and NPV, 1‐NPV, positivity rate and number of baseline colposcopies performed per disease case detected (1/PPV) were determined for each clinical endpoint (≥CIN2, ≥CIN3) for each HPV status, assessing triage using either DS or cytology both at baseline and using year‐1 cumulative disease results. These diagnostic measures were also calculated for partial genotyping scenarios, where only 12 “other” HPV‐positive cases were triaged with DS or cytology.

Sensitivity, specificity, PPV, NPV (1‐NPV) and positivity rates were reported as both fractions (n/N) and percentages. Two‐sided 95% confidence intervals (CIs) were calculated using (a) Wilson score method for sensitivity and specificity^20^; (b) score method according to Nam for PPV, NPV, 1‐NPV and 1/PPV^21^; (c) normal approximation for positivity rate; (d) Wilson score CI based method according to CLSI EP12‐A2 for differences in sensitivity and specificity^22^; and (e) percentile bootstrap method for differences in predictive values.^23^

There were no missing data for DS results, and unknown CPR reference diagnoses were not imputed. In disposition tables, the number of cases with unsatisfactory DS results is shown, and distributions for CPR results are shown for cases with both satisfactory and unsatisfactory DS results to enable assessment of potential bias.

A target sample size of 3500 HPV‐positive women was set in order for 95% CIs for 1‐NPV for co‐primary objectives to span ~3.2%. The obtained sample size of 5250 HPV‐positive women resulted in precision greater than planned.

## 3 RESULTS

### 3.1 Study and analysis populations

**FIGURE 1:**
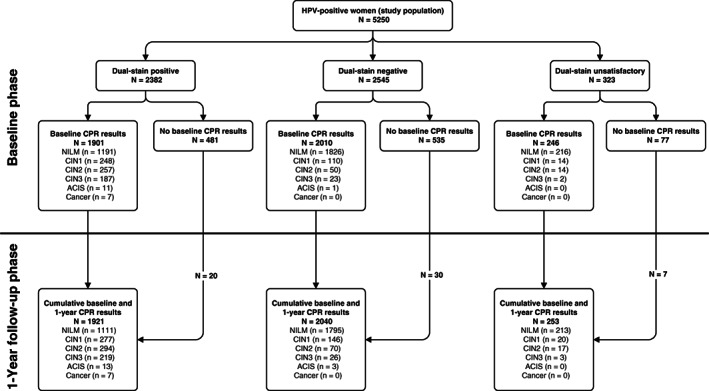
CONSORT diagram. Triage of cobas 6800/8800 HPV‐positive women. There were 481 (DS positive), 535 (DS negative) and 77 (DS unsatisfactory) cases with no baseline CPR results, which made them eligible to be referred to the 1 year follow‐up phase. Among them, there were 20, 30 and 7 cases in the DS positive, DS negative and DS unsatisfactory categories, respectively, which had valid 1‐year CPR results. ACIS, adenocarcinoma in situ; CIN, cervical intraepithelial neoplasia; CPR, central pathology review; DS, dual stain; NILM, negative for intraepithelial lesion or malignancy

A total of 5250 women with positive cobas 6800/8800 HPV test results at baseline were included in this analysis. For this cohort, the mean age at enrolment was 37.1 years (SD: 10.3), and median age was 34.0 years (range, 25.0‐65.0). The proportion of women aged 25 to 29 years was 29.9% (1568/5250). These and further study population characteristics and descriptive statistics are provided in Table S1. Figure 1 shows the analysis population of 4927 women with valid DS results and histologic endpoints at baseline as well as cumulative year‐1 follow‐up numbers.

### 3.2 DS positivity by cytology and biopsy results

Within the cobas 6800/8800 HPV‐positive study population with valid DS results, 536 women with ≥CIN2 were diagnosed at baseline and 632 women were diagnosed with ≥CIN2 cumulatively at baseline and/or year‐1 (Figure 1 *)*. Figure S2 provides the CONSORT diagram for cobas 4800 HPV‐positive women. In all, 2382 (48.3%) HPV‐positive women were positive for DS at baseline. DS positivity rates were 33.3% (1030/3090) in HPV‐positive women with cytologic NILM, and 62.8% (510/812), 79.0% (575/728), 90.8% (129/142) and 96.6% (114/118) in women with ASC‐US (atypical squamous cells of undetermined significance), LSIL, AGC/ASC‐H (atypical glandular cells/atypical squamous cells ‐ cannot rule out HSIL) and HSIL/ACIS (high‐grade squamous intraepithelial lesion/ACIS) cytology results, respectively (Table 1). Furthermore, DS positivity rates increased from 39.5% (1191/3017) of biopsy results (baseline CPR results) categorized as histological NILM to 69.3% (248/358) in CIN1, 83.7% (257/307) in CIN2, 89.0% (187/210) in CIN3, 91.7% (11/12) in ACIS and 100% (7/7) in invasive cervical cancer (Table 1). DS results by baseline cytology and cumulative 1‐year histology diagnoses are shown in Table S2. Tables S3 and S4 provide these results for cobas 4800 HPV‐positive women.

**TABLE 1:** HPV genotype and dual‐stain results by baseline cytology and baseline histology results among cobas 6800/8800 HPV‐positive women

	Baseline CPR histology result—subject n (%)							
Cytologic test result	Total (n = 4927)	Unavailable^a^ (n = 1016)	NILM (n = 3017)	CIN1 (n = 358)	CIN2 (n = 307)	CIN3 (n = 210)	ACIS (n = 12)	Cancer (n = 7)
**NILM**	**3090 (62.7)**	**668 (65.7)**	**2108 (69.9)**	**132 (36.9)**	**121 (39.4)**	**56 (26.7)**	**4 (33.3)**	**1 (14.3)**
HPV16+ and DS+	194 (33.2)	39 (32.2)	107 (27.2)	12 (50.0)	16 (84.2)	19 (73.1)	0	1 (100.0)
HPV16+ and DS−	391 (66.8)	82 (67.8)	287 (72.8)	12 (50.0)	3 (15.8)	7 (26.9)	0	0
HPV18+ and DS+	86 (27.7)	15 (24.2)	62 (27.0)	2 (22.2)	5 (71.4)	1 (100.0)	1 (50.0)	0
HPV18+ and DS−	225 (72.3)	47 (75.8)	168 (73.0)	7 (77.8)	2 (28.6)	0	1 (50.0)	0
12 other HPV+ and DS+	750 (34.2)	164 (33.8)	452 (30.5)	47 (47.5)	67 (70.5)	18 (62.1)	2 (100.0)	0
12 other HPV+ and DS−	1444 (65.8)	321 (66.2)	1032 (69.5)	52 (52.5)	28 (29.5)	11 (37.9)	0	0
**ASC‐US**	**812 (16.5)**	**144 (14.2)**	**487 (16.1)**	**86 (24.0)**	**63 (20.5)**	**31 (14.8)**	**1 (8.3)**	**0**
HPV16+ and DS+	113 (70.2)	25 (75.8)	55 (60.4)	8 (72.7)	12 (100.0)	12 (92.3)	1 (100.0)	0
HPV16+ and DS−	48 (29.8)	8 (24.2)	36 (39.6)	3 (27.3)	0	1 (7.7)	0	0
HPV18+ and DS+	36 (59.0)	10 (71.4)	18 (50.0)	3 (60.0)	3 (75.0)	2 (100.0)	0	0
HPV18+ and DS−	25 (41.0)	4 (28.6)	18 (50.0)	2 (40.0)	1 (25.0)	0	0	0
12 other HPV+ and DS+	361 (61.2)	60 (61.9)	191 (53.1)	55 (78.6)	41 (87.2)	14 (87.5)	0	0
12 other HPV+ and DS−	229 (38.8)	37 (38.1)	169 (46.9)	15 (21.4)	6 (12.8)	2 (12.5)	0	0
**AGC/ASC‐H**	**142 (2.9)**	**27 (2.7)**	**46 (1.5)**	**4 (1.1)**	**18 (5.9)**	**38 (18.1)**	**7 (58.3)**	**2 (28.6)**
HPV16+ and DS+	55 (93.2)	8 (88.9)	11 (84.6)	3 (100.0)	5 (100.0)	22 (95.7)	5 (100.0)	1 (100.0)
HP 16+ and DS−	4 (6.8)	1 (11.1)	2 (15.4)	0	0	1 (4.3)	0	0
HPV18+ and DS+	16 (94.1)	4 (100.0)	6 (85.7)	0	2 (100.0)	1 (100.0)	2 (100.0)	1 (100.0)
HPV18+ and DS−	1 (5.9)	0	1 (14.3)	0	0	0	0	0
12 other HPV+ and DS+	58 (87.9)	13 (92.9)	20 (76.9)	1 (100.0)	10 (90.9)	14 (100.0)	0	0
12 other HPV+ and DS−	8 (12.1)	1 (7.1)	6 (23.1)	0	1 (9.1)	0	0	0
**LSIL**	**728 (14.8)**	**150 (14.8)**	**333 (11.0)**	**129 (36.0)**	**84 (27.4)**	**31 (14.8)**	**0**	**1 (14.3)**
HPV16+ and DS+	121 (89.0)	22 (84.6)	41 (85.4)	16 (88.9)	26 (92.9)	16 (100.0)	0	0
HPV16+ and DS−	15 (11.0)	4 (15.4)	7 (14.6)	2 (11.1)	2 (7.1)	0	0	0
HPV18+ and DS+	44 (77.2)	8 (80.0)	15 (71.4)	13 (81.3)	6 (85.7)	2 (66.7)	0	0
HPV18+ and DS−	13 (22.8)	2 (20.0)	6 (28.6)	3 (18.8)	1 (14.3)	1 (33.3)	0	0
12 other HPV+ and DS+	410 (76.6)	89 (78.1)	181 (68.6)	81 (85.3)	46 (93.9)	12 (100.0)	0	1 (100.0)
12 other HPV+ and DS−	125 (23.4)	25 (21.9)	83 (31.4)	14 (14.7)	3 (6.1)	0	0	0
**HSIL/ACIS**	**118 (2.4)**	**15 (1.5)**	**23 (0.8)**	**5 (1.4)**	**18 (5.9)**	**54 (25.7)**	**0**	**3 (42.9)**
HPV16+ and DS+	51 (100.0)	3 (100.0)	7 (100.0)	1 (100.0)	6 (100.0)	32 (100.0)	0	2 (100.0)
HPV16+ and DS−	0	0	0	0	0	0	0	0
HPV18+ and DS+	10 (100.0)	2 (100.0)	2 (100.0)	1 (100.0)	1 (100.0)	3 (100.0)	0	1 (100.0)
HPV18+ and DS−	0	0	0	0	0	0	0	0
12 other HPV+ and DS+	53 (93.0)	9 (90.0)	12 (85.7)	3 (100.0)	10 (90.9)	19 (100.0)	0	0
12 other HPV+ and DS−	4 (7.0)	1 (10.0)	2 (14.3)	0	1 (9.1)	0	0	0
**Unavailable**	**37 (0.8)**	**12 (1.2)**	**20 (0.7)**	**2 (0.6)**	**3 (1.0)**	**0**	**0**	**0**
HPV16+ and DS+	6 (85.7)	4 (100.0)	1 (50.0)	0	1 (100.0)	0	0	0
HPV16+ and DS−	1 (14.3)	0	1 (50.0)	0	0	0	0	0
HPV18+ and DS+	3 (75.0)	2 (100.0)	1 (100.0)	0	0	0	0	0
HPV18+ and DS−	1 (25.0)	0	0	0	1 (100.0)	0	0	0
12 other HPV+ and DS+	15 (57.7)	4 (66.7)	9 (52.9)	2 (100.0)	0	0	0	0
12 other HPV+ and DS−	11 (42.3)	2 (33.3)	8 (47.1)	0	1 (100.0)	0	0	0
**Total**	**4927 (100.0)**	**1016 (100.0)**	**3017 (100.0)**	**358 (100.0)**	**307 (100.0)**	**210 (100.0)**	**12 (100.0)**	**7 (100.0)**
HPV16+ and DS+	540 (54.1)	101 (51.5)	222 (40.0)	40 (70.2)	66 (93.0)	101 (91.8)	6 (100.0)	4 (100.0)
HPV16+ and DS−	459 (45.9)	95 (48.5)	333 (60.0)	17 (29.8)	5 (7.0)	9 (8.2)	0	0
HPV18+ and DS+	195 (42.4)	41 (43.6)	104 (35.0)	19 (61.3)	17 (77.3)	9 (90.0)	3 (75.0)	2 (100.0)
HPV18+ and DS−	265 (57.6)	53 (56.4)	193 (65.0)	12 (38.7)	5 (22.7)	1 (10.0)	1 (25.0)	0
12 other HPV+ and DS+	1647 (47.5)	339 (46.7)	865 (40.0)	189 (70.0)	174 (81.3)	77 (85.6)	2 (100.0)	1 (100.0)
12 other HPV+ and DS−	1821 (52.5)	387 (53.3)	1300 (60.0)	81 (30.0)	40 (18.7)	13 (14.4)	0	0

*Note*: Percentages at the top rows of each cytology category (bolded) are column percentages with respect to that cytology category. Other percentages are column percentages with respect to the corresponding HPV genotype group.

Abbreviations: ACIS, adenocarcinoma in situ; AGC/ASC‐H, atypical glandular cells/atypical squamous cells—cannot exclude HSIL; ASC‐US, atypical squamous cells of undetermined significance; CIN, cervical intraepithelial neoplasia; CPR, central pathology review; DS, dual‐stain; HSIL, high‐grade squamous intraepithelial lesions; LSIL, low‐grade squamous intraepithelial lesions; NILM, negative for intraepithelial lesion or malignancy.

^a^ Out of 1016 cases with CPR results marked as “Unavailable,” 56 cases had inadequate CPR results, 143 cases had invalid CPR results due to the biopsies collected outside of the study visit window and 817 cases had no biopsy collected for CPR diagnosis.

### 3.3 DS performance in HPV16/18‐positive and 12 “other” HPV‐positive women

The performance of DS was assessed for the identification of high‐grade cervical disease (≥CIN2; ≥CIN3) when used to triage women aged 25 to 65 years with positive primary screening HPV test results, stratified by HPV16/18 vs 12 “other” genotype groups (Table 2).

**TABLE 2:** Triage of cobas 6800/8800 HPV‐positive women with dual‐stain and cytology by HPV genotype: baseline and cumulative year‐1 data for ≥CIN2 and ≥CIN3

			Sensitivity % (n/N) 95% CI		Specificity % (n/N) 95% CI		PPV % (n/N) 95% CI		1‐NPV % (n/N) 95% CI		Positivity rate % (n/N) 95% CI	Number of baseline colpos per disease detected % (n/N) 95% CI
Disease status	HPV status	Triage with	Baseline	Cumulative	Baseline	Cumulative	Baseline	Cumulative	Baseline	Cumulative	Baseline	Baseline
≥CIN2	HPV16/18+	DS	91.2 (207/227) (86.8, 94.2)	89.0 (234/263) (84.6, 92.2)	59.1 (554/937) (55.9, 62.2)	60.7 (556/916) (57.5, 63.8)	35.1 (207/590) (33.1, 37.1)	39.4 (234/594) (37.2, 41.6)	3.5 (20/574) (2.3, 5.2)	5.0 (29/585) (3.5, 6.8)	50.7 (590/1164) (48.1, 53.3)	2.85 (590/207) (2.70, 3.02)
		Cytology	75.3 (171/227) (69.3, 80.5)	73.0 (192/263) (67.3, 78.0)	70.1 (657/937) (67.1, 73.0)	71.3 (653/916) (68.3, 74.1)	37.9 (171/451) (35.0, 40.8)	42.2 (192/455) (39.1, 45.3)	7.9 (56/713) (6.3, 9.6)	9.8 (71/724) (8.1, 11.7)	38.7 (451/1164) (36.1, 41.3)	2.64 (451/171) (2.45, 2.86)
	HPV16+	DS	92.6 (176/190) (88.0, 95.6)	91.9 (193/210) (87.4, 94.9)	57.2 (349/610) (53.3, 61.1)	59.0 (356/603) (55.1, 62.9)	40.3 (176/437) (37.9, 42.7)	43.9 (193/440) (41.3, 46.5)	3.9 (14/363) (2.3, 6.2)	4.6 (17/373) (2.9, 6.9)	54.6 (437/800) (51.5, 57.7)	2.48 (437/176) (2.34, 2.64)
		Cytology	75.8 (144/190) (69.2, 81.3)	75.7 (159/210) (69.5, 81.0)	68.5 (418/610) (64.7, 72.1)	70.3 (424/603) (66.5, 73.8)	42.9 (144/336) (39.4, 46.3)	47.0 (159/338) (43.4, 50.7)	9.9 (46/464) (7.8, 12.3)	10.7 (51/475) (8.6, 13.2)	42.0 (336/800) (38.8, 45.2)	2.33 (336/144) (2.16, 2.54)
	HPV18+	DS	83.8 (31/37) (68.9, 92.3)	77.4 (41/53) (64.5, 86.5)	62.7 (205/327) (57.3, 67.8)	63.9 (200/313) (58.4, 69.0)	20.3 (31/153) (16.8, 23.4)	26.6 (41/154) (22.4, 30.6)	2.8 (6/211) (1.4, 5.4)	5.7 (12/212) (3.4, 8.7)	42.0 (153/364) (37.2, 46.9)	4.94 (153/31) (4.27, 5.96)
		Cytology	73.0 (27/37) (57.0, 84.6)	62.3 (33/53) (48.8, 74.1)	73.1 (239/327) (68.0, 77.6)	73.2 (229/313) (68.0, 77.8)	23.5 (27/115) (18.6, 28.2)	28.2 (33/117) (22.6, 33.8)	4.0 (10/249) (2.3, 6.3)	8.0 (20/249) (5.6, 10.7)	31.6 (115/364) (27.0, 36.2)	4.26 (115/27) (3.55, 5.38)
	12 other HPV+	DS	83.0 (254/306) (78.4, 86.8)	81.4 (298/366) (77.1, 85.1)	56.8 (1373/2416) (54.8, 58.8)	57.5 (1376/2391) (55.6, 59.5)	19.6 (254/1297) (18.5, 20.6)	22.7 (298/1313) (21.5, 23.9)	3.6 (52/1425) (2.9, 4.6)	4.7 (68/1444) (3.8, 5.8)	47.6 (1297/2722) (45.8, 49.5)	5.11 (1297/254) (4.85, 5.41)
		Cytology	58.8 (180/306) (53.2, 64.2)	57.7 (211/366) (52.5, 62.6)	65.5 (1583/2416) (63.6, 67.4)	66.0 (1577/2391) (64.0, 67.8)	17.8 (180/1013) (16.2, 19.3)	20.6 (211/1025) (18.9, 22.3)	7.4 (126/1709) (6.5, 8.3)	8.9 (155/1732) (8.0, 10.0)	37.2 (1013/2722) (35.4, 39.0)	5.63 (1013/180) (5.17, 6.18)
≥CIN3	HPV16/18+	DS	91.9 (125/136) (86.1, 95.4)	91.0 (142/156) (85.5, 94.6)	54.8 (563/1028) (51.7, 57.8)	55.8 (571/1023) (52.8, 58.8)	21.2 (125/590) (19.7, 22.6)	23.9 (142/594) (22.3, 25.4)	1.9 (11/574) (1.1, 3.3)	2.4 (14/585) (1.5, 3.8)	50.7 (590/1164) (47.9, 53.4)	4.72 (590/125) (4.43, 5.07)
		Cytology	77.9 (106/136) (70.3, 84.1)	75.6 (118/156) (68.3, 81.7)	66.4 (683/1028) (63.5, 69.3)	67.1 (686/1023) (64.1, 69.9)	23.5 (106/451) (21.2, 25.7)	25.9 (118/455) (23.5, 28.3)	4.2 (30/713) (3.1, 5.6)	5.2 (38/724) (4.0, 6.7)	38.7 (451/1164) (36.1, 41.4)	4.25 (451/106) (3.89, 4.71)
	HPV16+	DS	92.5 (111/120) (86.4, 96.0)	92.4 (122/132) (86.6, 95.8)	52.1 (354/680) (48.3, 55.8)	53.3 (363/681) (49.5, 57.0)	25.4 (111/437) (23.6, 27.2)	27.7 (122/440) (25.8, 29.6)	2.5 (9/363) (1.3, 4.4)	2.7 (10/373) (1.5, 4.7)	54.6 (437/800) (51.4, 57.9)	3.94 (437/111) (3.68, 4.24)
		Cytology	77.5 (93/120) (69.2, 84.1)	76.5 (101/132) (68.6, 82.9)	64.3 (437/680) (60.6, 67.8)	65.2 (444/681) (61.5, 68.7)	27.7 (93/336) (24.8, 30.5)	29.9 (101/338) (26.9, 32.8)	5.8 (27/464) (4.2, 7.8)	6.5 (31/475) (4.8, 8.6)	42.0 (336/800) (38.7, 45.3)	3.61 (336/93) (3.28, 4.03)
	HPV18+	DS	87.5 (14/16) (64.0, 96.5)	83.3 (20/24) (64.1, 93.3)	60.1 (209/348) (54.8, 65.1)	60.8 (208/342) (55.6, 65.8)	9.2 (14/153) (6.7, 10.8)	13.0 (20/154) (10.1, 15.3)	0.9 (2/211) (0.3, 2.7)	1.9 (4/212) (0.8, 4.0)	42.0 (153/364) (37.1, 47.0)	10.93 (153/14) (9.29, 14.83)
		Cytology	81.3 (13/16) (57.0, 93.4)	70.8 (17/24) (50.8, 85.1)	70.7 (246/348) (65.7, 75.2)	70.8 (242/342) (65.7, 75.3)	11.3 (13/115) (8.0, 13.9)	14.5 (17/117) (10.6, 18.1)	1.2 (3/249) (0.4, 2.7)	2.8 (7/249) (1.5, 4.7)	31.6 (115/364) (26.9, 36.2)	8.85 (115/13) (7.20, 12.50)
	12 other HPV+	DS	86.0 (80/93) (77.5, 91.6)	86.6 (97/112) (79.1, 91.7)	53.7 (1412/2629) (51.8, 55.6)	54.0 (1429/2645) (52.1, 55.9)	6.2 (80/1297) (5.6, 6.6)	7.4 (97/1313) (6.7, 7.9)	0.9 (13/1425) (0.5, 1.5)	1.0 (15/1444) (0.6, 1.6)	47.6 (1297/2722) (45.8, 49.5)	16.21 (1297/80) (15.07, 17.98)
		Cytology	66.7 (62/93) (56.6, 75.4)	65.2 (73/112) (56.0, 73.4)	63.8 (1678/2629) (62.0, 65.6)	64.0 (1693/2645) (62.2, 65.8)	6.1 (62/1013) (5.2, 6.9)	7.1 (73/1025) (6.1, 8.0)	1.8 (31/1709) (1.3, 2.4)	2.3 (39/1732) (1.7, 2.8)	37.2 (1013/2722) (35.4, 39.0)	16.34 (1013/62) (14.40, 19.19)

Abbreviations: CI, confidence interval; CIN, cervical intraepithelial neoplasia; DS, dual‐stain; NPV, negative predictive value; PPV, positive predictive value.

In HPV16/18‐positive women, DS sensitivity for ≥CIN2 and ≥CIN3 at baseline was 91.2% and 91.9%, respectively, and specificity was 59.1% for ≥CIN2 and 54.8% for ≥CIN3. PPV of DS positivity was high in HPV16/18‐positive women, reaching 35.1% for ≥CIN2 and 21.2% for ≥CIN3 at baseline. The risk of disease in HPV16/18‐positive, DS‐negative women (1‐NPV) for ≥CIN3 at baseline were 1.9%, meeting one of the prespecified acceptance criteria for the co‐primary study objective of the trial (1‐NPV: ≤5.0% for ≥CIN3). Overall, similar sensitivity, specificity, PPV and 1‐NPV estimates were observed for cumulative vs baseline disease endpoints, that is, for ≥CIN2 (≥CIN3) detected at baseline and/or after the 1‐year follow‐up (Table 2).

In 12 “other” HPV‐positive women, sensitivity of DS for ≥CIN2 and ≥CIN3 at baseline was 83.0% and 86.0%, respectively, significantly higher as compared to the respective sensitivity estimates of cytology: 58.8% for ≥CIN2 and 66.7% for ≥CIN3 (Table 2). DS showed lower specificity but similar to slightly higher PPV for ≥CIN2 as compared to cytology in the triage of 12 “other” HPV‐positive women. However, the rate of disease in test negatives (1‐NPV) for ≥CIN2 was significantly lower in DS negative women (3.6%) compared to cytology negative, 12 “other” HPV‐positive women (7.4%; *P* < .0001), cutting the number to less than half (Table 2). Table S5 provides these results for cobas 4800 HPV‐positive women.

### 3.4 DS vs cytology, alone or combined with HPV16/18 genotyping, for detecting high‐grade CIN

DS alone showed a significantly higher sensitivity for the detection of ≥CIN2 in HPV‐positive women at baseline than cytology combined with HPV16/18 genotyping (86.5% vs 76.4%; *P* < .0001) or cytology alone (65.9%; *P* < .0001) (Table 3). Similar results were observed at the ≥CIN3 disease threshold, and for cumulative year‐1 data. Specificity of DS alone was significantly higher than specificity of HPV16/18 genotyping combined with cytology (for ≥CIN2 at baseline, 57.5% vs 47.2%; *P* < .0001), but significantly lower than observed for cytology alone (66.8%; *P* < .0001). Of note, triage with DS alone would have referred significantly fewer women to colposcopy than HPV16/18 genotyping with cytology triage for 12 “other” HPV‐positive women (48.6% vs 56.0%; *P* < .0001), leading to significantly higher efficiency as shown by the lower number of 4.09 vs 5.35; *P* < .0001) colposcopies to be performed per ≥CIN2 detected (Table 3). Adding HPV16/18 genotyping to DS provided the highest sensitivity (90.2% for ≥CIN2 and 94.3% for ≥CIN3 at baseline), however, at the cost of a substantially lower specificity compared to DS alone (Table 3). Table S6 provides these results for cobas 4800 HPV‐positive women.

**TABLE 3:** Triage performance of dual‐stain and cytology, alone or in combination with HPV16/18 genotyping for detecting ≥CIN2 and ≥CIN3 in cobas 6800/8800 HPV‐positive women: baseline and cumulative 1‐year data

Disease status	Triage with	Sensitivity % (n/N) 95% CI		Specificity % (n/N) 95% CI		PPV % (n/N) 95% CI		1‐NPV % (n/N) 95% CI		Positivity rate % (n/N) 95% CI	Number of baseline colpos per disease detected % (n/N) 95% CI
		Baseline	Cumulative	Baseline	Cumulative	Baseline	Cumulative	Baseline	Cumulative	Baseline	Baseline
≥CIN2	DS	86.5 (461/533) (83.3, 89.1)	84.6 (532/629) (81.5, 87.2)	57.5 (1927/3353) (55.8, 59.1)	58.4 (1932/3307) (56.7, 60.1)	24.4 (461/1887) (23.5, 25.4)	27.9 (532/1907) (26.8, 28.9)	3.6 (72/1999) (2.9, 4.4)	4.8 (97/2029) (4.0, 5.7)	48.6 (1887/3886) (47.1, 50.1)	4.09 (1887/461) (3.94, 4.26)
	Cytology	65.9 (351/533) (61.7, 69.8)	64.1 (403/629) (60.2, 67.7)	66.8 (2240/3353) (65.2, 68.4)	67.4 (2230/3307) (65.8, 69.0)	24.0 (351/1464) (22.6, 25.4)	27.2 (403/1480) (25.7, 28.7)	7.5 (182/2422) (6.7, 8.4)	9.2 (226/2456) (8.3, 10.1)	37.7 (1464/3886) (36.2, 39.2)	4.17 (1464/351) (3.94, 4.43)
	HPV16/18 GT + DS	90.2 (481/533) (87.4, 92.5)	89.2 (561/629) (86.5, 91.4)	40.9 (1373/3353) (39.3, 42.6)	41.6 (1376/3307) (39.9, 43.3)	19.5 (481/2461) (18.9, 20.2)	22.5 (561/2492) (21.8, 23.2)	3.6 (52/1425) (2.8, 4.7)	4.7 (68/1444) (3.8, 5.8)	63.3 (2461/3886) (61.9, 64.8)	5.12 (2461/481) (4.96, 5.29)
	HPV16/18 GT + cytology	76.4 (407/533) (72.6, 79.8)	75.4 (474/629) (71.8, 78.6)	47.2 (1583/3353) (45.5, 48.9)	47.7 (1577/3307) (46.0, 49.4)	18.7 (407/2177) (17.8, 19.5)	21.5 (474/2204) (20.6, 22.4)	7.4 (126/1709) (6.4, 8.5)	8.9 (155/1732) (7.8, 10.1)	56.0 (2177/3886) (54.5, 57.6)	5.35 (2177/407) (5.12, 5.62)
≥CIN3	DS	89.5 (205/229) (84.9, 92.9)	89.2 (239/268) (84.9, 92.4)	54.0 (1975/3657) (52.4, 55.6)	54.5 (2000/3668) (52.9, 56.1)	10.9 (205/1887) (10.3, 11.4)	12.5 (239/1907) (11.9, 13.1)	1.2 (24/1999) (0.8, 1.7)	1.4 (29/2029) (1.0, 2.0)	48.6 (1887/3886) (47.0, 50.1)	9.20 (1887/205) (8.79, 9.73)
	Cytology	73.4 (168/229) (67.3, 78.7)	71.3 (191/268) (65.6, 76.4)	64.6 (2361/3657) (63.0, 66.1)	64.9 (2379/3668) (63.3, 66.4)	11.5 (168/1464) (10.5, 12.4)	12.9 (191/1480) (11.9, 13.9)	2.5 (61/2422) (2.0, 3.1)	3.1 (77/2456) (2.6, 3.7)	37.7 (1464/3886) (36.2, 39.2)	8.71 (1464/168) (8.10, 9.49)
	HPV16/18 GT + DS	94.3 (216/229) (90.5, 96.7)	94.4 (253/268) (91.0, 96.6)	38.6 (1412/3657) (37.0, 40.2)	39.0 (1429/3668) (37.4, 40.5)	8.8 (216/2461) (8.4, 9.1)	10.2 (253/2492) (9.8, 10.5)	0.9 (13/1425) (0.5, 1.5)	1.0 (15/1444) (0.6, 1.7)	63.3 (2461/3886) (61.8, 64.8)	11.39 (2461/216) (11.02, 11.89)
	HPV16/18 GT + cytology	86.5 (198/229) (81.4, 90.3)	85.4 (229/268) (80.7, 89.2)	45.9 (1678/3657) (44.3, 47.5)	46.2 (1693/3668) (44.5, 47.8)	9.1 (198/2177) (8.6, 9.6)	10.4 (229/2204) (9.8, 10.9)	1.8 (31/1709) (1.3, 2.5)	2.3 (39/1732) (1.7, 3.0)	56.0 (2177/3886) (54.5, 57.6)	10.99 (2177/198) (10.47, 11.68)

Abbreviations: CI, confidence interval; CIN, cervical intraepithelial neoplasia; DS, dual‐stain; GT, genotypes; NPV, negative predictive value; PPV, positive predictive value.

### 3.5 Risk of high‐grade CIN in HPV‐positive women with positive or negative triage test results

**FIGURE 2:**
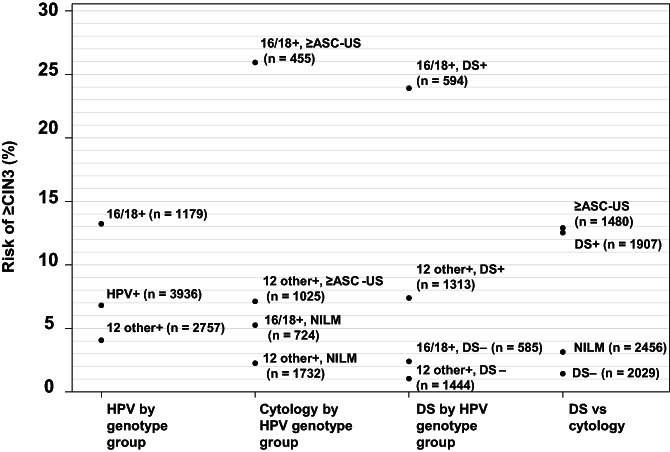
Risk of ≥CIN3 in cobas 6800/8800 HPV‐positive women dependent on HPV genotype group, cytology and dual‐stain results. Cumulative 1‐year risks of ≥CIN3 for HPV genotype groups (16/18+; 12 other+), cytology (NILM, ≥ASC‐US) and dual‐stain (DS+, DS−) test results are plotted on the *y*‐axis, with the number of subjects within the respective categories indicated

The risk of ≥CIN2 and ≥CIN3 among HPV‐positive women for the various triage strategies using DS or cytology, either combined with HPV16/18 genotyping or alone, is provided in Table 3 and graphically presented in Figure 2 for ≥CIN3. Results for cobas 4800 HPV‐positive women are provided in Table S6 and Figure S3. HPV‐positive women with negative DS test results showed a very low cumulative 1‐year risk for disease (1‐NPV for ≥CIN3: 1.4%), significantly lower than the respective risks when using cytology with HPV16/18 genotyping (2.3%; *P* = .0181), or cytology alone (3.1%; *P* < .0001) (Table 3). A similar level of reduction of the cumulative 1‐year risk for disease was observed at the ≥CIN2 threshold, that is, 1‐NPV of 4.8% for DS vs 8.9% and 9.2% for cytology combined with HPV16/18 genotyping and cytology alone, respectively. DS provided a better risk stratification than cytology combined with HPV16/18 genotyping, identifying a larger number of women with very low risk for ≥CIN3 (2029 DS negative women; 51.5%) as compared to combined cytology/HPV16/18 genotyping (1732 women with NILM/12 “other” HPV‐positive results; 44.0%), whereas less women would be referred to colposcopy (1887 vs 2177). A DS negative result consistently showed the lowest risk for ≥CIN3 across all triage strategies.

Women with HPV16/18‐positive and DS positive results had the highest risk for ≥CIN3, whereas the risk was lowest in women with 12 “other” HPV‐positive women with negative DS results. Of note, the risk for ≥CIN3 was similar in HPV16/18‐positive women with negative DS results as in 12 “other” HPV‐positive women with NILM (Figure 2).

## 4 DISCUSSION

Many countries have either transitioned or are in the process of transitioning from cytology‐based cervical cancer screening to primary HPV screening. Primary HPV screening has a high sensitivity for detecting ≥CIN3 lesions, but has a low specificity, especially in young women who frequently have transient HPV infections. Therefore, additional triage is needed to identify HPV‐positive women at greatest risk for ≥CIN3. One approach that has been endorsed by various professional societies and used in the United States since 2014 is HPV16/18 genotyping with cytology triage of 12 “other” HPV‐positive women.^1^, ^24^ Another promising approach is DS cytology. DS has been previously shown to provide high sensitivity and specificity when used for cervical cancer screening,^11^ as a triage of women with equivocal or mildly abnormal cervical cytology^25^ and as a triage of HPV‐positive women.^9^, ^10^, ^13^

One of the main objectives of the IMPACT trial was to evaluate the clinical performance of DS as a triage for HPV‐positive women undergoing primary HPV screening either by itself or in combination with HPV16/18 genotyping. DS provided both high sensitivity and good specificity for the detection of either ≥CIN2 or ≥CIN3 in HPV‐positive women. Replacing cytology with DS as the triage for women with 12 “other” genotypes in the current primary HPV screening algorithm which includes HPV16/18 genotyping resulted in a significant increase in sensitivity for ≥CIN3 and a modest reduction in specificity. Although the colposcopy rate at baseline using DS triage increased from 56.0% to 63.3%, because more cases of ≥CIN3 were detected using DS, the number of colposcopies needed to detect a single case of ≥CIN3 was similar (10.99 vs 11.39, respectively). Similar to the results seen in women with 12 “other” genotypes, DS‐negative, HPV16/18‐positive women also had a lower risk of ≥CIN3 than those who were cytology negative, HPV16/18 positive. Similar performance estimates were observed for cross‐sectional data analysis using the baseline colposcopy data and after a 1‐year follow‐up period, and DS triage met the prespecified primary study objectives of the IMPACT trial.

The comparative performance of DS vs cytology in the current trial is similar to what was previously reported from the ATHENA study, but differs from what was reported in a 3‐year follow‐up study of HPV‐positive women from Kaiser Permanente Northern California (KPNC).^9^, ^13^, ^15^ In the ATHENA study, replacing cytology with DS as the triage for women with 12 “other” genotypes in the algorithm with HPV16/18 genotyping resulted in a significant increase in sensitivity for ≥CIN3 detected at baseline (86.8% and 78.2%, respectively) but similar specificities (57.4% and 57.6%, respectively).^13^ In contrast, the KPNC study found no significant difference in sensitivity for ≥CIN3 when HPV16/18 genotyping with cytology triage of 12 “other” genotypes was used compared to HPV16/18 genotyping with DS triage of 12 “other” genotypes (92.8% vs 92.4%, respectively). Triage of 12 “other” genotypes with DS also had a significantly higher specificity (46.5%) compared to cytology (36.1%). There are several differences between the KPNC study and IMPACT that could potentially explain why the results differ. One is that HPV‐positive women in the KPNC study were managed according to standard clinical guidelines. Women with negative cytology underwent repeat co‐testing at 1 year, irrespective of HPV genotype, and women only received colposcopy if the repeat test was positive. Another difference is that cytology in the KPNC study had an especially high sensitivity and a low specificity.^9^ Furthermore, two HPV tests were utilized in IMPACT, and women positive on either HPV test or cytology were referred to colposcopy. In the KPNC study, the sensitivity of cytology for ≥CIN3 (3 years, cumulative) in HPV‐positive women, irrespective of genotype, was 84.3% and specificity was 42.9%. In IMPACT, the sensitivity of cytology for ≥CIN3 (1 year, cumulative) in HPV‐positive women, irrespective of genotype, was 71.3% and specificity was 64.9%. The ATHENA study had a similar study design as IMPACT and the sensitivity of cytology for ≥CIN3 (baseline) in HPV‐positive women, irrespective of genotype, was 52.8% and specificity was 64.9%.^26^ It is important to note that in contrast to the variable performance of cytology in the KPNC study and IMPACT, the performance of DS in the two studies was highly consistent. The sensitivity for ≥CIN3 of the algorithm incorporating HPV16/18 genotyping with DS triage of 12 “other” genotypes was 94.3% (1 year, cumulative) in IMPACT and 92.4% (3 years, cumulative) in KPNC.

Since the risk of ≥CIN2 or ≥CIN3 in DS‐negative, HPV‐positive women was low, irrespective of HPV genotype, we evaluated the performance of DS as a stand‐alone triage test for HPV‐positive women. DS used as the sole triage tool for HPV‐positive women provided significantly better sensitivity and specificity than the current algorithm of HPV16/18 genotyping and cytology triage of 12 “other” genotypes. When DS is used alone to triage HPV‐positive women the cumulative risk (1‐NPV) of ≥CIN3 in triage‐negative women was only 1.4% compared to 2.3% in women who were triage negative using the algorithm of HPV16/18 genotyping with cytology triage of 12 “other” genotypes. Triaging HPV‐positive women with DS alone would have referred a significantly lower number of women to colposcopy vs HPV16/18 genotyping with cytology triage of 12 “other” genotypes (48.6% vs 56.0%, respectively; *P* < .0001). Similar findings were found in the 3‐year KPNC study.^9^ The 3‐year risk of ≥CIN3 in HPV‐positive, DS‐negative women was 1.7% compared to 1.4% in triage‐negative women using HPV16/18 genotyping and cytology for 12 “other” genotypes. Another KPNC study evaluated the long‐term reassurance that a negative DS result provides in HPV‐positive women.^10^ DS‐negative, HPV‐positive women had a lower 5‐year risk of ≥CIN2 than cytology‐negative, HPV‐positive women. Even after 5 years, the risk of ≥CIN3 remained below KPNC's colposcopy referral threshold.

Since risk of ≥CIN3 was consistently lowest whenever DS was negative, irrespective of HPV genotype, DS generally provides the best risk stratification for HPV‐positive women. Current American Society for Colposcopy and Cervical Pathology (ASCCP) guidelines take a risk‐centered approach to patient management based on a woman's risk of ≥CIN3.^27^, ^28^ A key risk threshold is ≥4% immediate risk of ≥CIN3 which is the risk level at which women are referred to colposcopy. Women at lower risk of ≥CIN3 can undergo either 12‐month follow‐up or interval screening. Irrespective of whether HPV‐positive women are triaged using an algorithm incorporating HPV16/18 genotyping and DS for 12 “other” genotypes or triaged using DS alone, the risk of ≥CIN3 in DS triage‐negative women is considerably less than 4%. Even in HPV16/18‐positive women the risk of ≥CIN3 does not meet the colposcopy referral threshold if they are DS negative. The length of follow‐up of the current study was limited to 1 year. However, two other studies from KPNC have reported similar results with up to 5 years of follow‐up.^9^, ^10^

Another activity that utilizes considerable screening resources is retesting triage‐negative women at 12 months. Risk cutoffs for returning women to routine screening vary. The ASCCP recommends that only women with ≤0.55% 5‐year risk of ≥CIN3 return to routine screening.^27^, ^28^ At this risk threshold, none of the HPV‐positive women in our study could return to routine screening regardless of triage approach. However, the two KPNC studies of DS used a different risk threshold.^9^, ^10^ Their threshold was the risk of ≥CIN3 in HPV‐positive women with negative cytology. The 1‐year risk of ≥CIN3 in these women was 2.8%.^9^ Using the KPNC risk threshold, women with 12 “other” genotypes who are cytology or DS negative as well as DS negative, HPV positive (without genotyping) in the KPNC studies and IMPACT could return to routine screening.

Our study has several strengths and limitations. Strengths include that IMPACT was a large prospective study that enrolled women in 32 clinical centers and assessed DS test performance in 4 central laboratories that also performed the cytology and HPV testing. Disease ascertainment was maximized by referring all women who tested positive with either of two HPV tests or who had an abnormal cytology to colposcopy. Women who fulfilled the initial colposcopy referral criteria were followed‐up at 1 year, and those who were either HPV or cytology positive at 1 year were referred for another colposcopy. To eliminate potential study bias, colposcopy was performed blinded to all test results and a nontargeted biopsy was collected when no lesion was identified at colposcopy. A CPR was performed on both H&E and H&E + p16‐stained biopsy specimens. Limitations include the fact that the study follow‐up was limited to 1 year and therefore the assessment of the negative disease prediction of a negative DS for a period longer than 1 year cannot be made.

In conclusion, the results of the IMPACT trial demonstrate that DS is safe and effective for the triage of HPV‐positive women identified during primary HPV screening. DS alone or in combination with HPV16/18 genotyping offers an alternative to current triage strategies which are based on cytology, either alone or combined with HPV16/18 genotyping. DS‐based triage provides consistently higher sensitivity than cytology‐based triage, providing better reassurance against ≥CIN2 and ≥CIN3. Using DS alone as the triage reduces the complexity of triage strategies for HPV‐positive women.

## CONFLICT OF INTEREST

Drs. Stoler and Wright are consultants to Roche, BD Life Sciences, Inovio, and QSquared Solutions. They are speakers for Roche and BD Life Sciences. The other authors are employees of Roche.

## ETHICS STATEMENT

All patients enrolled into the trial provided their informed consent before any study procedures. The study protocol and all amendments were approved by an Institutional Review Board (IRB). The trial was conducted in compliance with International Conference on Harmonization (ICH) Good Clinical Practice (GCP) Guidelines, applicable regulations of the U.S. Food and Drug Administration (FDA), and in accordance with the ethical principles originating in the Declaration of Helsinki. This is an observational, noninterventional diagnostic study and in alignment with FDAAA2007, World Health Organization (WHO) and ICMJE was not registered with ClinicalTrials.gov.

## Supporting information


**Supplementary Figure 1** Flow of subjects through baseline and follow‐up phases of the IMPACT trial
**Supplementary Figure 2.** CONSORT diagram. Triage of cobas 4800 HPV‐positive women
**Supplementary Figure 3.** Risk of ≥CIN3 in cobas 4800 HPV‐positive women dependent on HPV genotype group, cytology, and Dual‐stain results
**Supplementary Table 1**. Demographic characteristics and HPV vaccination status by Dual‐stain results of the cobas 6800/8800 HPV‐positive study population
**Supplementary Table 2.** HPV genotype and Dual‐stain results by baseline cytology and cumulative 1‐year histology results among cobas 6800/8800 HPV‐positive women
**Supplementary Table 3.** HPV genotype and Dual‐stain results by cytology and baseline histology results among cobas 4800 HPV‐positive women
**Supplementary Table 4.** HPV genotype and Dual‐stain results by baseline cytology and cumulative 1‐year histology results among cobas 4800 HPV‐positive women
**Supplementary Table 5.** Triage of cobas 4800 HPV‐positive women with Dual‐stain and cytology by HPV genotype group: baseline and cumulative 1‐year data for ≥CIN2 and ≥ CIN3
**Supplementary Table 6.** Triage performance of Dual‐stain and cytology, alone or in combination with HPV16/18 genotyping for detecting ≥CIN2 and ≥ CIN3 in cobas 4800 HPV‐positive women: baseline and cumulative 1‐year data

## Data Availability

The data that support the findings of this study are available from the corresponding author upon reasonable request.
