# Supplementary material for: Clinical validation of p16/Ki‐67 dual‐stained cytology triage of HPV‐positive women: Results from the IMPACT trial
Source: Int J Cancer. 2021 Sep 25;150(3):461–71. doi: 10.1002/ijc.33812 (PMC9293341; doi:10.1002/ijc.33812)
Supplement: Supplementary file 1 — Supplementary Figure 1 Flow of subjects through baseline and follow‐up phases of the IMPACT trial Supplementary Figure 2. CONSORT diagram. Triage of cobas 4800 HPV‐positive women Supplementary Figure 3. Risk of ≥CIN3 in cobas 4800 HPV‐positive women dependent on HPV genotype group, cytology, and Dual‐stain results Supplementary Table 1. Demographic characteristics and HPV vaccination status by Dual‐stain results of the cobas 6800/8800 HPV‐positive study population Supplementary Table 2. HPV genotype and Dual‐stain results by baseline cytology and cumulative 1‐year histology results among cobas 6800/8800 HPV‐positive women Supplementary Table 3. HPV genotype and Dual‐stain results by cytology and baseline histology results among cobas 4800 HPV‐positive women Supplementary Table 4. HPV genotype and Dual‐stain results by baseline cytology and cumulative 1‐year histology results among cobas 4800 HPV‐positive women Supplementary Table 5. Triage of cobas 4800 HPV‐positive women with Dual‐stain and cytology by HPV genotype group: baseline and cumulative 1‐year data for ≥CIN2 and ≥ CIN3 Supplementary Table 6. Triage performance of Dual‐stain and cytology, alone or in combination with HPV16/18 genotyping for detecting ≥CIN2 and ≥ CIN3 in cobas 4800 HPV‐positive women: baseline and cumulative 1‐year data [file IJC-150-461-s001.pdf]

Clinical Validation of p16/Ki-67 Dual-Stained Cytology Triage of HPV-Positive Women – Results from the IMPACT Trial

Wright Jr. TC, Stoler MH, Ranger-Moore J, Fang Q, Volkir P, Safaeian M, Ridder R.

Supplementary Materials

Supplementary Table 1. Demographic characteristics and HPV vaccination status by Dual-stain results of the cobas 6800/8800 HPV-positive study population

Supplementary Table 2. HPV genotype and Dual-stain results by baseline cytology and cumulative 1-year histology results among cobas 6800/8800 HPV-positive women

Supplementary Table 3. HPV genotype and Dual-stain results by cytology and baseline histology results among cobas 4800 HPV-positive women

Supplementary Table 4. HPV genotype and Dual-stain results by baseline cytology and cumulative 1-year histology results among cobas 4800 HPV-positive women

Supplementary Table 5. Triage of cobas 4800 HPV-positive women with Dual-stain and cytology by HPV genotype group: baseline and cumulative 1-year data for ≥CIN2 and ≥CIN3

Supplementary Table 6. Triage performance of Dual-stain and cytology, alone or in combination with HPV16/18 genotyping for detecting ≥CIN2 and ≥CIN3 in cobas 4800 HPV-positive women: baseline and cumulative 1-year data

Supplementary Figure 1. Flow of subjects through baseline and follow-up phases of the IMPACT trial

Supplementary Figure 2. CONSORT diagram. Triage of cobas 4800 HPV-positive women

Supplementary Figure 3. Risk of ≥CIN3 in cobas 4800 HPV-positive women dependent on HPV genotype group, cytology, and Dual-stain results

Supplementary Table 1. Demographic characteristics and HPV vaccination status by Dual-stain results of the cobas 6800/8800 HPV-positive study population

	**Dual-stain Results**			
**Demographic Characteristic**	**Positive (N=2382)**	**Negative (N=2545)**	**Invalid (N=323)**	**Total (N=5250)**
**Age**				
Mean (SD)	36.5 (10.4)	37.5 (10.2)	37.5 (10.5)	37.1 (10.3)
Median	33.0	35.0	34.0	34.0
Min, Max	25.0, 65.0	25.0, 65.0	25.0, 64.0	25.0, 65.0
**Age group**				
25-29	776 (49.5)	700 (44.6)	92 (5.9)	1568 (29.9)
30-39	884 (45.5)	941 (48.4)	119 (6.1)	1944 (37.0)
40-49	351 (38.4)	501 (54.8)	62 (6.8)	914 (17.4)
50-59	278 (43.9)	320 (50.6)	35 (5.5)	633 (12.1)
60-65	93 (48.7)	83 (43.5)	15 (7.9)	191 (3.6)
**Ethnicity**				
Hispanic or Latino	672 (49.7)	619 (45.8)	62 (4.6)	1353 (25.8)
Not Hispanic or Latino	1688 (43.9)	1897 (49.4)	257 (6.7)	3842 (73.2)
Unknown	9 (45.0)	11 (55.0)	0 (0.0)	20 (0.4)
Not Reported	13 (37.1)	18 (51.4)	4 (11.4)	35 (0.7)
**Race**				
American Indian/Alaskan Native	12 (40.0)	15 (50.0)	3 (10.0)	30 (0.6)
Asian	46 (43.8)	47 (44.8)	12 (11.4)	105 (2.0)
Black/African-American	571 (42.1)	687 (50.7)	97 (7.2)	1355 (25.8)
Native Hawaiian/Pacific Islander	6 (46.2)	7 (53.8)	0 (0.0)	13 (0.2)
White	1653 (46.9)	1682 (47.7)	193 (5.5)	3528 (67.2)
Other	62 (41.1)	78 (51.7)	11 (7.3)	151 (2.9)
Unknown/Not Reported	32 (47.1)	29 (42.6)	7 (10.3)	68 (1.3)
**Vaccination status**				
HPV Vaccine				
No	1997 (45.4)	2130 (48.4)	275 (6.2)	4402 (83.8)
Missing	1 (100.0)	0 (0.0)	0 (0.0)	1 (0.0)
Yes	384 (45.3)	415 (49.0)	48 (5.7)	847 (16.1)
Type of vaccine				
Gardasil	305 (45.1)	333 (49.3)	38 (5.6)	676 (79.8)
Gardasil 9	9 (60.0)	6 (40.0)	0 (0.0)	15 (1.8)
Cervarix	1 (50.0)	1 (50.0)	0 (0.0)	2 (0.2)
Unknown	69 (44.8)	75 (48.7)	10 (6.5)	154 (18.2)

Invalid Dual-stain results include unsatisfactory test results and negative test results with invalid run controls.
Row percentage is calculated for each Dual-stain category, column percentage is calculated for total

Supplementary Table 2. HPV genotype and Dual-stain results by baseline cytology and cumulative 1-year histology results among cobas 6800/8800 HPV-positive women

	Cumulative 1-Year CPR Histology Result − Subject n (%)							
**Cytologic Test Result**	**Total (n=4927)**	**Unavailable* (n=966)**	**NILM (n=2906)**	**CIN1 (n=423)**	**CIN2 (n=364)**	**CIN3 (n=245)**	**ACIS (n=16)**	**Cancer (n=7)**
**NILM**	**3090 (62.7)**	**634 (65.6)**	**2061 (70.9)**	**169 (40.0)**	**149 (40.9)**	**69 (28.2)**	**7 (43.8)**	**1 (14.3)**
HPV16+ and DS+	194 (33.2)	36 (32.7)	105 (26.7)	14 (45.2)	16 (80.0)	22 (73.3)	0	1 (100.0)
HPV16+ and DS-	391 (66.8)	74 (67.3)	288 (73.3)	17 (54.8)	4 (20.0)	8 (26.7)	0	0
HPV18+ and DS+	86 (27.7)	15 (24.2)	56 (26.0)	4 (28.6)	7 (53.8)	2 (100.0)	2 (40.0)	0
HPV18+ and DS-	225 (72.3)	47 (75.8)	159 (74.0)	10 (71.4)	6 (46.2)	0	3 (60.0)	0
12 Other HPV+ and DS+	750 (34.2)	156 (33.8)	434 (29.9)	57 (46.0)	77 (66.4)	24 (64.9)	2 (100.0)	0
12 Other HPV+ and DS-	1444 (65.8)	306 (66.2)	1019 (70.1)	67 (54.0)	39 (33.6)	13 (35.1)	0	0
**ASC-US**	**812 (16.5)**	**138 (14.3)**	**461 (15.9)**	**103 (24.3)**	**70 (19.2)**	**38 (15.5)**	**2 (12.5)**	**0**
HPV16+ and DS+	113 (70.2)	25 (78.1)	49 (58.3)	10 (71.4)	13 (92.9)	14 (93.3)	2 (100.0)	0
HPV16+ and DS-	48 (29.8)	7 (21.9)	35 (41.7)	4 (28.6)	1 (7.1)	1 (6.7)	0	0
HPV18+ and DS+	36 (59.0)	10 (76.9)	16 (45.7)	4 (66.7)	3 (75.0)	3 (100.0)	0	0
HPV18+ and DS-	25 (41.0)	3 (23.1)	19 (54.3)	2 (33.3)	1 (25.0)	0	0	0
12 Other HPV+ and DS+	361 (61.2)	58 (62.4)	181 (52.9)	60 (72.3)	44 (84.6)	18 (90.0)	0	0
12 Other HPV+ and DS-	229 (38.8)	35 (37.6)	161 (47.1)	23 (27.7)	8 (15.4)	2 (10.0)	0	0
**AGC/ASC-H**	**142 (2.9)**	**27 (2.8)**	**35 (1.2)**	**7 (1.7)**	**24 (6.6)**	**40 (16.3)**	**7 (43.8)**	**2 (28.6)**
HPV16+ and DS+	55 (93.2)	8 (88.9)	6 (75.0)	4 (100.0)	8 (100.0)	23 (95.8)	5 (100.0)	1 (100.0)
HPV16+ and DS-	4 (6.8)	1 (11.1)	2 (25.0)	0	0	1 (4.2)	0	0
HPV18+ and DS+	16 (94.1)	4 (100.0)	4 (80.0)	1 (100.0)	2 (100.0)	2 (100.0)	2 (100.0)	1 (100.0)
HPV18+ and DS-	1 (5.9)	0	1 (20.0)	0	0	0	0	0
12 Other HPV+ and DS+	58 (87.9)	13 (92.9)	16 (72.7)	2 (100.0)	13 (92.9)	14 (100.0)	0	0
12 Other HPV+ and DS-	8 (12.1)	1 (7.1)	6 (27.3)	0	1 (7.1)	0	0	0
**LSIL**	**728 (14.8)**	**140 (14.5)**	**310 (10.7)**	**135 (31.9)**	**99 (27.2)**	**43 (17.6)**	**0**	**1 (14.3)**
HPV16+ and DS+	121 (89.0)	22 (88.0)	34 (87.2)	17 (77.3)	28 (93.3)	20 (100.0)	0	0
HPV16+ and DS-	15 (11.0)	3 (12.0)	5 (12.8)	5 (22.7)	2 (6.7)	0	0	0
HPV18+ and DS+	44 (77.2)	7 (77.8)	13 (68.4)	12 (80.0)	8 (88.9)	4 (80.0)	0	0
HPV18+ and DS-	13 (22.8)	2 (22.2)	6 (31.6)	3 (20.0)	1 (11.1)	1 (20.0)	0	0
12 Other HPV+ and DS+	410 (76.6)	83 (78.3)	169 (67.1)	83 (84.7)	56 (93.3)	18 (100.0)	0	1 (100.0)
12 Other HPV+ and DS-	125 (23.4)	23 (21.7)	83 (32.9)	15 (15.3)	4 (6.7)	0	0	0

Cytologic Test Result	Cumulative 1-Year CPR Histology Result − Subject n (%)							
	Total (n=4927)	Unavailable* (n=966)	NILM (n=2906)	CIN1 (n=423)	CIN2 (n=364)	CIN3 (n=245)	ACIS (n=16)	Cancer (n=7)
**HSIL/ACIS**	**118 (2.4)**	**15 (1.6)**	**19 (0.7)**	**7 (1.7)**	**19 (5.2)**	**55 (22.4)**	**0**	**3 (42.9)**
HPV16+ and DS+	51 (100.0)	3 (100.0)	6 (100.0)	2 (100.0)	6 (100.0)	32 (100.0)	0	2 (100.0)
HPV16+ and DS-	0	0	0	0	0	0	0	0
HPV18+ and DS+	10 (100.0)	2 (100.0)	2 (100.0)	1 (100.0)	1 (100.0)	3 (100.0)	0	1 (100.0)
HPV18+ and DS-	0	0	0	0	0	0	0	0
12 Other HPV+ and DS+	53 (93.0)	9 (90.0)	9 (81.8)	4 (100.0)	11 (91.7)	20 (100.0)	0	0
12 Other HPV+ and DS-	4 (7.0)	1 (10.0)	2 (18.2)	0	1 (8.3)	0	0	0
**Unavailable**	**37 (0.8)**	**12 (1.2)**	**20 (0.7)**	**2 (0.5)**	**3 (0.8)**	**0**	**0**	**0**
HPV16+ and DS+	6 (85.7)	4 (100.0)	1 (50.0)	0	1 (100.0)	0	0	0
HPV16+ and DS-	1 (14.3)	0	1 (50.0)	0	0	0	0	0
HPV18+ and DS+	3 (75.0)	2 (100.0)	1 (100.0)	0	0	0	0	0
HPV18+ and DS-	1 (25.0)	0	0	0	1 (100.0)	0	0	0
12 Other HPV+ and DS+	15 (57.7)	4 (66.7)	9 (52.9)	2 (100.0)	0	0	0	0
12 Other HPV+ and DS-	11 (42.3)	2 (33.3)	8 (47.1)	0	1 (100.0)	0	0	0
**Total**	**4927 (100.0)**	**966 (100.0)**	**2906 (100.0)**	**423 (100.0)**	**364 (100.0)**	**245 (100.0)**	**16 (100.0)**	**7 (100.0)**
HPV16+ and DS+	540 (54.1)	98 (53.6)	201 (37.8)	47 (64.4)	72 (91.1)	111 (91.7)	7 (100.0)	4 (100.0)
HPV16+ and DS-	459 (45.9)	85 (46.4)	331 (62.2)	26 (35.6)	7 (8.9)	10 (8.3)	0	0
HPV18+ and DS+	195 (42.4)	40 (43.5)	92 (33.2)	22 (59.5)	21 (70.0)	14 (93.3)	4 (57.1)	2 (100.0)
HPV18+ and DS-	265 (57.6)	52 (56.5)	185 (66.8)	15 (40.5)	9 (30.0)	1 (6.7)	3 (42.9)	0
12 Other HPV+ and DS+	1647 (47.5)	323 (46.7)	818 (39.0)	208 (66.5)	201 (78.8)	94 (86.2)	2 (100.0)	1 (100.0)
12 Other HPV+ and DS-	1821 (52.5)	368 (53.3)	1279 (61.0)	105 (33.5)	54 (21.2)	15 (13.8)	0	0

*Out of 966 cases with CPR results marked as "Unavailable", 38 cases had inadequate CPR results, 111 cases had invalid CPR results due to the biopsies collected outside of the study visit window, and 817 cases had no biopsy collected for CPR diagnosis.

Percentages at the top rows of each cytology category (bolded) are column percentages with respect to that cytology category. Other percentages are column percentages with respect to the corresponding HPV genotype group. DS, Dual-stain; CPR, central pathology review; NILM, negative for intraepithelial lesion or malignancy; ASC-US, atypical squamous cells of undetermined significance; AGC/ASC-H, atypical glandular cells/atypical squamous cells - cannot exclude HSIL; LSIL, low-grade squamous intraepithelial lesions; HSIL, high-grade squamous intraepithelial lesions; ACIS, adenocarcinoma in-situ; CIN, cervical intraepithelial neoplasia

Supplementary Table 3. HPV genotype and Dual-stain results by cytology and baseline histology results among cobas 4800 HPV-positive women

	Baseline CPR Histology Result − Subject n (%)							
**Cytologic Test Result**	**Total (n=4727)**	**Unavailable* (n=975)**	**NILM (n=2864)**	**CIN1 (n=352)**	**CIN2 (n=310)**	**CIN3 (n=208)**	**ACIS (n=11)**	**Cancer (n=7)**
**NILM**	**2901 (61.4)**	**631 (64.7)**	**1964 (68.6)**	**125 (35.5)**	**122 (39.4)**	**54 (26.0)**	**4 (36.4)**	**1 (14.3)**
HPV16+ and DS+	167 (46.1)	33 (44.6)	86 (38.2)	12 (63.2)	16 (84.2)	19 (79.2)	0	1 (100.0)
HPV16+ and DS-	195 (53.9)	41 (55.4)	139 (61.8)	7 (36.8)	3 (15.8)	5 (20.8)	0	0
HPV18+ and DS+	54 (36.0)	9 (40.9)	36 (32.1)	2 (28.6)	5 (83.3)	1 (100.0)	1 (50.0)	0
HPV18+ and DS-	96 (64.0)	13 (59.1)	76 (67.9)	5 (71.4)	1 (16.7)	0	1 (50.0)	0
12 Other HPV+ and DS+	775 (32.4)	175 (32.7)	467 (28.7)	48 (48.5)	65 (67.0)	18 (62.1)	2 (100.0)	0
12 Other HPV+ and DS-	1614 (67.6)	360 (67.3)	1160 (71.3)	51 (51.5)	32 (33.0)	11 (37.9)	0	0
**ASC-US**	**792 (16.8)**	**137 (14.1)**	**473 (16.5)**	**87 (24.7)**	**63 (20.3)**	**31 (14.9)**	**1 (9.1)**	**0**
HPV16+ and DS+	99 (75.6)	24 (82.8)	44 (64.7)	5 (71.4)	13 (100.0)	12 (92.3)	1 (100.0)	0
HPV16+ and DS-	32 (24.4)	5 (17.2)	24 (35.3)	2 (28.6)	0	1 (7.7)	0	0
HPV18+ and DS+	25 (61.0)	7 (77.8)	10 (47.6)	4 (57.1)	2 (100.0)	2 (100.0)	0	0
HPV18+ and DS-	16 (39.0)	2 (22.2)	11 (52.4)	3 (42.9)	0	0	0	0
12 Other HPV+ and DS+	372 (60.0)	63 (63.6)	195 (50.8)	57 (78.1)	43 (89.6)	14 (87.5)	0	0
12 Other HPV+ and DS-	248 (40.0)	36 (36.4)	189 (49.2)	16 (21.9)	5 (10.4)	2 (12.5)	0	0
**AGC/ASC-H**	**141 (3.0)**	**26 (2.7)**	**46 (1.6)**	**4 (1.1)**	**19 (6.1)**	**38 (18.3)**	**6 (54.5)**	**2 (28.6)**
HPV16+ and DS+	55 (96.5)	8 (100.0)	12 (92.3)	3 (100.0)	5 (100.0)	21 (95.5)	5 (100.0)	1 (100.0)
HPV16+ and DS-	2 (3.5)	0	1 (7.7)	0	0	1 (4.5)	0	0
HPV18+ and DS+	15 (100.0)	4 (100.0)	6 (100.0)	0	2 (100.0)	1 (100.0)	1 (100.0)	1 (100.0)
HPV18+ and DS-	0	0	0	0	0	0	0	0
12 Other HPV+ and DS+	60 (87.0)	13 (92.9)	21 (77.8)	1 (100.0)	10 (83.3)	15 (100.0)	0	0
12 Other HPV+ and DS-	9 (13.0)	1 (7.1)	6 (22.2)	0	2 (16.7)	0	0	0
**LSIL**	**733 (15.5)**	**153 (15.7)**	**335 (11.7)**	**129 (36.6)**	**84 (27.1)**	**31 (14.9)**	**0**	**1 (14.3)**
HPV16+ and DS+	118 (88.1)	22 (81.5)	40 (85.1)	14 (87.5)	26 (92.9)	16 (100.0)	0	0
HPV16+ and DS-	16 (11.9)	5 (18.5)	7 (14.9)	2 (12.5)	2 (7.1)	0	0	0
HPV18+ and DS+	44 (81.5)	9 (90.0)	14 (73.7)	14 (87.5)	5 (83.3)	2 (66.7)	0	0
HPV18+ and DS-	10 (18.5)	1 (10.0)	5 (26.3)	2 (12.5)	1 (16.7)	1 (33.3)	0	0
12 Other HPV+ and DS+	419 (76.9)	89 (76.7)	187 (69.5)	83 (85.6)	47 (94.0)	12 (100.0)	0	1 (100.0)
12 Other HPV+ and DS-	126 (23.1)	27 (23.3)	82 (30.5)	14 (14.4)	3 (6.0)	0	0	0

Cytologic Test Result	Baseline CPR Histology Result − Subject n (%) Total (n=4727)	Unavailable* (n=975)	NILM (n=2864)	CIN1 (n=352)	CIN2 (n=310)	CIN3 (n=208)	ACIS (n=11)	Cancer (n=7)
**HSIL/ACIS**	**118 (2.5)**	**15 (1.5)**	**23 (0.8)**	**5 (1.4)**	**18 (5.8)**	**54 (26.0)**	**0**	**3 (42.9)**
HPV16+ and DS+	51 (100.0)	3 (100.0)	7 (100.0)	1 (100.0)	6 (100.0)	32 (100.0)	0	2 (100.0)
HPV16+ and DS-	0	0	0	0	0	0	0	0
HPV18+ and DS+	10 (100.0)	2 (100.0)	2 (100.0)	1 (100.0)	1 (100.0)	3 (100.0)	0	1 (100.0)
HPV18+ and DS-	0	0	0	0	0	0	0	0
12 Other HPV+ and DS+	53 (93.0)	9 (90.0)	12 (85.7)	3 (100.0)	10 (90.9)	19 (100.0)	0	0
12 Other HPV+ and DS-	4 (7.0)	1 (10.0)	2 (14.3)	0	1 (9.1)	0	0	0
**Unavailable**	**42 (0.9)**	**13 (1.3)**	**23 (0.8)**	**2 (0.6)**	**4 (1.3)**	**0**	**0**	**0**
HPV16+ and DS+	6 (85.7)	4 (100.0)	1 (50.0)	0	1 (100.0)	0	0	0
HPV16+ and DS-	1 (14.3)	0	1 (50.0)	0	0	0	0	0
HPV18+ and DS+	1 (50.0)	1 (100.0)	0	0	0	0	0	0
HPV18+ and DS-	1 (50.0)	0	0	0	1 (100.0)	0	0	0
12 Other HPV+ and DS+	17 (51.5)	5 (62.5)	9 (42.9)	2 (100.0)	1 (50.0)	0	0	0
12 Other HPV+ and DS-	16 (48.5)	3 (37.5)	12 (57.1)	0	1 (50.0)	0	0	0
**Total**	**4727 (100.0)**	**975 (100.0)**	**2864 (100.0)**	**352 (100.0)**	**310 (100.0)**	**208 (100.0)**	**11 (100.0)**	**7 (100.0)**
HPV16+ and DS+	496 (66.8)	94 (64.8)	190 (52.5)	35 (76.1)	67 (93.1)	100 (93.5)	6 (100.0)	4 (100.0)
HPV16+ and DS-	246 (33.2)	51 (35.2)	172 (47.5)	11 (23.9)	5 (6.9)	7 (6.5)	0	0
HPV18+ and DS+	149 (54.8)	32 (66.7)	68 (42.5)	21 (67.7)	15 (83.3)	9 (90.0)	2 (66.7)	2 (100.0)
HPV18+ and DS-	123 (45.2)	16 (33.3)	92 (57.5)	10 (32.3)	3 (16.7)	1 (10.0)	1 (33.3)	0
12 Other HPV+ and DS+	1696 (45.7)	354 (45.3)	891 (38.0)	194 (70.5)	176 (80.0)	78 (85.7)	2 (100.0)	1 (100.0)
12 Other HPV+ and DS-	2017 (54.3)	428 (54.7)	1451 (62.0)	81 (29.5)	44 (20.0)	13 (14.3)	0	0

*Out of 975 cases with CPR results marked as "Unavailable", 55 cases had inadequate CPR results, 136 cases had invalid CPR results due to the biopsies collected outside of the study visit window, and 784 cases had no biopsy collected for CPR diagnosis.

Percentages at the top rows of each cytology category (bolded) are column percentages with respect to that cytology category. Other percentages are column percentages with respect to the corresponding HPV genotype group. DS, Dual-stain; CPR, central pathology review; NILM, negative for intraepithelial lesion or malignancy; ASC-US, atypical squamous cells of undetermined significance; AGC/ASC-H, atypical glandular cells/atypical squamous cells - cannot exclude HSIL; LSIL, low-grade squamous intraepithelial lesions; HSIL, high-grade squamous intraepithelial lesions; ACIS, adenocarcinoma in-situ; CIN, cervical intraepithelial neoplasia

Supplementary Table 4. HPV genotype and Dual-stain results by baseline Pap cytology and cumulative 1-year histology results among cobas 4800 HPV-positive women

Cytologic Test Result	Cumulative 1-Year CPR Histology Result − Subject n (%)							
	**Total (n=4727)**	**Unavailable* (n=933)**	**NILM (n=2749)**	**CIN1 (n=415)**	**CIN2 (n=366)**	**CIN3 (n=243)**	**ACIS (n=14)**	**Cancer (n=7)**
**NILM**	**2901 (61.4)**	**605 (64.8)**	**1914 (69.6)**	**159 (38.3)**	**150 (41.0)**	**67 (27.6)**	**5 (35.7)**	**1 (14.3)**
HPV16+ and DS+	167 (46.1)	31 (44.3)	83 (37.7)	13 (56.5)	17 (85.0)	22 (78.6)	0	1 (100.0)
HPV16+ and DS-	195 (53.9)	39 (55.7)	137 (62.3)	10 (43.5)	3 (15.0)	6 (21.4)	0	0
HPV18+ and DS+	54 (36.0)	9 (40.9)	31 (30.4)	4 (40.0)	7 (63.6)	2 (100.0)	1 (33.3)	0
HPV18+ and DS-	96 (64.0)	13 (59.1)	71 (69.6)	6 (60.0)	4 (36.4)	0	2 (66.7)	0
12 Other HPV+ and DS+	775 (32.4)	168 (32.7)	449 (28.2)	58 (46.0)	74 (62.2)	24 (64.9)	2 (100.0)	0
12 Other HPV+ and DS-	1614 (67.6)	345 (67.3)	1143 (71.8)	68 (54.0)	45 (37.8)	13 (35.1)	0	0
**ASC-US**	**792 (16.8)**	**132 (14.1)**	**447 (16.3)**	**105 (25.3)**	**69 (18.9)**	**38 (15.6)**	**1 (7.1)**	**0**
HPV16+ and DS+	99 (75.6)	24 (85.7)	40 (63.5)	7 (70.0)	13 (92.9)	14 (93.3)	1 (100.0)	0
HPV16+ and DS-	32 (24.4)	4 (14.3)	23 (36.5)	3 (30.0)	1 (7.1)	1 (6.7)	0	0
HPV18+ and DS+	25 (61.0)	7 (77.8)	8 (42.1)	5 (62.5)	2 (100.0)	3 (100.0)	0	0
HPV18+ and DS-	16 (39.0)	2 (22.2)	11 (57.9)	3 (37.5)	0	0	0	0
12 Other HPV+ and DS+	372 (60.0)	61 (64.2)	185 (50.7)	62 (71.3)	46 (86.8)	18 (90.0)	0	0
12 Other HPV+ and DS-	248 (40.0)	34 (35.8)	180 (49.3)	25 (28.7)	7 (13.2)	2 (10.0)	0	0
**AGC/ASC-H**	**141 (3.0)**	**26 (2.8)**	**33 (1.2)**	**7 (1.7)**	**25 (6.8)**	**40 (16.5)**	**8 (57.1)**	**2 (28.6)**
HPV16+ and DS+	55 (96.5)	8 (100.0)	7 (87.5)	4 (100.0)	8 (100.0)	22 (95.7)	5 (100.0)	1 (100.0)
HPV16+ and DS-	2 (3.5)	0	1 (12.5)	0	0	1 (4.3)	0	0
HPV18+ and DS+	15 (100.0)	4 (100.0)	3 (100.0)	1 (100.0)	2 (100.0)	2 (100.0)	2 (100.0)	1 (100.0)
HPV18+ and DS-	0	0	0	0	0	0	0	0
12 Other HPV+ and DS+	60 (87.0)	13 (92.9)	16 (72.7)	2 (100.0)	13 (86.7)	15 (100.0)	1 (100.0)	0
12 Other HPV+ and DS-	9 (13.0)	1 (7.1)	6 (27.3)	0	2 (13.3)	0	0	0
**LSIL**	**733 (15.5)**	**142 (15.2)**	**313 (11.4)**	**135 (32.5)**	**99 (27.0)**	**43 (17.7)**	**0**	**1 (14.3)**
HPV16+ and DS+	118 (88.1)	22 (84.6)	33 (84.6)	15 (78.9)	28 (93.3)	20 (100.0)	0	0
HPV16+ and DS-	16 (11.9)	4 (15.4)	6 (15.4)	4 (21.1)	2 (6.7)	0	0	0
HPV18+ and DS+	44 (81.5)	8 (88.9)	12 (70.6)	13 (86.7)	7 (87.5)	4 (80.0)	0	0
HPV18+ and DS-	10 (18.5)	1 (11.1)	5 (29.4)	2 (13.3)	1 (12.5)	1 (20.0)	0	0
12 Other HPV+ and DS+	419 (76.9)	83 (77.6)	175 (68.1)	85 (84.2)	57 (93.4)	18 (100.0)	0	1 (100.0)
12 Other HPV+ and DS-	126 (23.1)	24 (22.4)	82 (31.9)	16 (15.8)	4 (6.6)	0	0	0

Cytologic Test Result	Total (n=4727)	Unavailable* (n=933)	NILM (n=2749)	CIN1 (n=415)	CIN2 (n=366)	CIN3 (n=243)	ACIS (n=14)	Cancer (n=7)
	Cumulative 1-Year CPR Histology Result − Subject n (%)							
**HSIL/ACIS**	**118 (2.5)**	**15 (1.6)**	**19 (0.7)**	**7 (1.7)**	**19 (5.2)**	**55 (22.6)**	**0**	**3 (42.9)**
HPV16+ and DS+	51 (100.0)	3 (100.0)	6 (100.0)	2 (100.0)	6 (100.0)	32 (100.0)	0	2 (100.0)
HPV16+ and DS-	0	0	0	0	0	0	0	0
HPV18+ and DS+	10 (100.0)	2 (100.0)	2 (100.0)	1 (100.0)	1 (100.0)	3 (100.0)	0	1 (100.0)
HPV18+ and DS-	0	0	0	0	0	0	0	0
12 Other HPV+ and DS+	53 (93.0)	9 (90.0)	9 (81.8)	4 (100.0)	11 (91.7)	20 (100.0)	0	0
12 Other HPV+ and DS-	4 (7.0)	1 (10.0)	2 (18.2)	0	1 (8.3)	0	0	0
**Unavailable**	**42 (0.9)**	**13 (1.4)**	**23 (0.8)**	**2 (0.5)**	**4 (1.1)**	**0**	**0**	**0**
HPV16+ and DS+	6 (85.7)	4 (100.0)	1 (50.0)	0	1 (100.0)	0	0	0
HPV16+ and DS-	1 (14.3)	0	1 (50.0)	0	0	0	0	0
HPV18+ and DS+	1 (50.0)	1 (100.0)	0	0	0	0	0	0
HPV18+ and DS-	1 (50.0)	0	0	0	1 (100.0)	0	0	0
12 Other HPV+ and DS+	17 (51.5)	5 (62.5)	9 (42.9)	2 (100.0)	1 (50.0)	0	0	0
12 Other HPV+ and DS-	16 (48.5)	3 (37.5)	12 (57.1)	0	1 (50.0)	0	0	0
**Total**	**4727 (100.0)**	**933 (100.0)**	**2749 (100.0)**	**415 (100.0)**	**366 (100.0)**	**243 (100.0)**	**14 (100.0)**	**7 (100.0)**
HPV16+ and DS+	496 (66.8)	92 (66.2)	170 (50.3)	41 (70.7)	73 (92.4)	110 (93.2)	6 (100.0)	4 (100.0)
HPV16+ and DS-	246 (33.2)	47 (33.8)	168 (49.7)	17 (29.3)	6 (7.6)	8 (6.8)	0	0
HPV18+ and DS+	149 (54.8)	31 (66.0)	56 (39.2)	24 (68.6)	19 (76.0)	14 (93.3)	3 (60.0)	2 (100.0)
HPV18+ and DS-	123 (45.2)	16 (34.0)	87 (60.8)	11 (31.4)	6 (24.0)	1 (6.7)	2 (40.0)	0
12 Other HPV+ and DS+	1696 (45.7)	339 (45.4)	843 (37.2)	213 (66.1)	202 (77.1)	95 (86.4)	3 (100.0)	1 (100.0)
12 Other HPV+ and DS-	2017 (54.3)	408 (54.6)	1425 (62.8)	109 (33.9)	60 (22.9)	15 (13.6)	0	0

*Out of 933 cases with CPR results marked as "Unavailable", 37 cases had inadequate CPR results, 112 cases had invalid CPR results due to the biopsies collected outside of the study visit window, and 784 cases had no biopsy collected for CPR diagnosis.

Percentages at the top rows of each cytology category (bolded) are column percentages with respect to that cytology category. Other percentages are column percentages with respect to the corresponding HPV genotype group. DS, Dual-stain; CPR, central pathology review; NILM, negative for intraepithelial lesion or malignancy; ASC-US, atypical squamous cells of undetermined significance; AGC/ASC-H, atypical glandular cells/atypical squamous cells - cannot exclude HSIL; LSIL, low-grade squamous intraepithelial lesions; HSIL, high-grade squamous intraepithelial lesions; ACIS, adenocarcinoma in-situ; CIN, cervical intraepithelial neoplasia

Supplementary Table 5. Triage of cobas 4800 HPV-positive women with Dual-stain and cytology by HPV genotype group: baseline and cumulative 1-year data for ≥CIN2 and ≥CIN3

			Sensitivity<br>%<br>(n/N)<br>95%CI		Specificity<br>%<br>(n/N)<br>95%CI		PPV<br>%<br>(n/N)<br>95%CI		1-NPV<br>%<br>(n/N)<br>95%CI		Positivity rate<br>%<br>(n/N)<br>95%CI	Number of baseline colpos per disease detected<br>%<br>(n/N)<br>95%CI
**Disease status**	**HPV Status**	**Triage with**	**Baseline**	**Cumulative**	**Baseline**	**Cumulative**	**Baseline**	**Cumulative**	**Baseline**	**Cumulative**	**Baseline**	**Baseline**
**≥CIN2**	HPV16/18+	DS	92.7<br>(204/220)<br>(88.5, 95.5)	91.3<br>(230/252)<br>(87.1, 94.2)	47.6<br>(284/597)<br>(43.6, 51.6)	49.3<br>(282/572)<br>(45.2, 53.4)	39.5<br>(204/517)<br>(37.5, 41.5)	44.2<br>(230/520)<br>(42.1, 46.5)	5.3<br>(16/300)<br>(3.4, 8.2)	7.2<br>(22/304)<br>(4.9, 10.4)	63.3<br>(517/817)<br>(60.2, 66.3)	2.53<br>(517/204)<br>(2.41, 2.67)
		Cytology	75.9<br>(167/220)<br>(69.8, 81.1)	74.2<br>(187/252)<br>(68.5, 79.2)	60.8<br>(363/597)<br>(56.8, 64.6)	62.1<br>(355/572)<br>(58.0, 65.9)	41.6<br>(167/401)<br>(38.6, 44.7)	46.3<br>(187/404)<br>(43.1, 49.5)	12.7<br>(53/416)<br>(10.2, 15.6)	15.5<br>(65/420)<br>(12.8, 18.4)	49.1<br>(401/817)<br>(45.8, 52.3)	2.40<br>(401/167)<br>(2.24, 2.59)
	HPV16+	DS	93.6<br>(176/188)<br>(89.2, 96.3)	93.2<br>(192/206)<br>(88.9, 95.9)	44.8<br>(182/406)<br>(40.1, 49.7)	46.7<br>(184/394)<br>(41.8, 51.6)	44.0<br>(176/400)<br>(41.7, 46.4)	47.8<br>(192/402)<br>(45.3, 50.3)	6.2<br>(12/194)<br>(3.6, 10.2)	7.1<br>(14/198)<br>(4.3, 11.2)	67.3<br>(400/594)<br>(63.9, 70.8)	2.27<br>(400/176)<br>(2.15, 2.40)
		Cytology	76.6<br>(144/188)<br>(70.0, 82.1)	76.2<br>(157/206)<br>(70.0, 81.5)	60.1<br>(244/406)<br>(55.3, 64.7)	61.7<br>(243/394)<br>(56.8, 66.3)	47.1<br>(144/306)<br>(43.5, 50.6)	51.0<br>(157/308)<br>(47.3, 54.7)	15.3<br>(44/288)<br>(12.0, 19.0)	16.8<br>(49/292)<br>(13.4, 20.5)	51.5<br>(306/594)<br>(47.7, 55.3)	2.13<br>(306/144)<br>(1.97, 2.30)
	HPV18+	DS	87.5<br>(28/32)<br>(71.9, 95.0)	82.6<br>(38/46)<br>(69.3, 90.9)	53.4<br>(102/191)<br>(46.3, 60.3)	55.1<br>(98/178)<br>(47.7, 62.2)	23.9<br>(28/117)<br>(19.9, 27.6)	32.2<br>(38/118)<br>(27.5, 36.8)	3.8<br>(4/106)<br>(1.5, 8.2)	7.5<br>(8/106)<br>(4.0, 12.8)	52.5<br>(117/223)<br>(46.2, 58.7)	4.18<br>(117/28)<br>(3.63, 5.02)
		Cytology	71.9<br>(23/32)<br>(54.6, 84.4)	65.2<br>(30/46)<br>(50.8, 77.3)	62.3<br>(119/191)<br>(55.3, 68.9)	62.9<br>(112/178)<br>(55.6, 69.7)	24.2<br>(23/95)<br>(18.8, 29.3)	31.3<br>(30/96)<br>(25.1, 37.3)	7.0<br>(9/128)<br>(4.0, 11.0)	12.5<br>(16/128)<br>(8.4, 17.1)	42.6<br>(95/223)<br>(36.3, 48.9)	4.13<br>(95/23)<br>(3.41, 5.31)
	12 Other HPV+	DS	82.1<br>(256/312)<br>(77.4, 85.9)	80.2<br>(300/374)<br>(75.9, 83.9)	58.6<br>(1520/2594)<br>(56.7, 60.5)	59.3<br>(1522/2567)<br>(57.4, 61.2)	19.2<br>(256/1330)<br>(18.1, 20.3)	22.3<br>(300/1345)<br>(21.1, 23.5)	3.6<br>(56/1576)<br>(2.8, 4.4)	4.6<br>(74/1596)<br>(3.8, 5.6)	45.8<br>(1330/2906)<br>(44.0, 47.5)	5.20<br>(1330/256)<br>(4.93, 5.51)
		Cytology	59.0<br>(184/312)<br>(53.4, 64.3)	57.8<br>(216/374)<br>(52.7, 62.7)	66.5<br>(1726/2594)<br>(64.7, 68.3)	66.9<br>(1718/2567)<br>(65.1, 68.7)	17.5<br>(184/1052)<br>(15.9, 19.0)	20.3<br>(216/1065)<br>(18.6, 21.9)	6.9<br>(128/1854)<br>(6.0, 7.8)	8.4<br>(158/1876)<br>(7.5, 9.4)	36.2<br>(1052/2906)<br>(34.5, 37.9)	5.72<br>(1052/184)<br>(5.26, 6.28)

Disease status	HPV Status	Triage with	Sensitivity<br>%<br>(n/N)<br>95%CI		Specificity<br>%<br>(n/N)<br>95%CI		PPV<br>%<br>(n/N)<br>95%CI		1-NPV<br>%<br>(n/N)<br>95%CI		Positivity rate<br>%<br>(n/N)<br>95%CI	Number of baseline colpos per disease detected<br>%<br>(n/N)<br>95%CI
			**Baseline**	**Cumulative**	**Baseline**	**Cumulative**	**Baseline**	**Cumulative**	**Baseline**	**Cumulative**	**Baseline**	**Baseline**
**≥CIN3**	HPV16/18+	DS	93.2<br>(123/132)<br>(87.5, 96.4)	92.7<br>(139/150)<br>(87.3, 95.9)	42.5<br>(291/685)<br>(38.8, 46.2)	43.5<br>(293/674)<br>(39.8, 47.2)	23.8<br>(123/517)<br>(22.3, 25.2)	26.7<br>(139/520)<br>(25.1, 28.3)	3.0<br>(9/300)<br>(1.6, 5.4)	3.6<br>(11/304)<br>(2.1, 6.1)	63.3<br>(517/817)<br>(60.1, 66.5)	4.20<br>(517/123)<br>(3.96, 4.49)
		Cytology	78.8<br>(104/132)<br>(71.1, 84.9)	77.3<br>(116/150)<br>(70.0, 83.3)	56.6<br>(388/685)<br>(52.9, 60.3)	57.3<br>(386/674)<br>(53.5, 61.0)	25.9<br>(104/401)<br>(23.5, 28.3)	28.7<br>(116/404)<br>(26.1, 31.2)	6.7<br>(28/416)<br>(4.9, 9.0)	8.1<br>(34/420)<br>(6.1, 10.5)	49.1<br>(401/817)<br>(45.8, 52.4)	3.86<br>(401/104)<br>(3.54, 4.25)
	HPV16+	DS	94.0<br>(110/117)<br>(88.2, 97.1)	93.8<br>(120/128)<br>(88.2, 96.8)	39.2<br>(187/477)<br>(34.9, 43.7)	40.3<br>(190/472)<br>(35.9, 44.7)	27.5<br>(110/400)<br>(25.7, 29.2)	29.9<br>(120/402)<br>(28.0, 31.7)	3.6<br>(7/194)<br>(1.8, 7.0)	4.0<br>(8/198)<br>(2.1, 7.5)	67.3<br>(400/594)<br>(63.7, 71.0)	3.64<br>(400/110)<br>(3.42, 3.88)
		Cytology	78.6<br>(92/117)<br>(70.4, 85.1)	77.3<br>(99/128)<br>(69.4, 83.7)	55.1<br>(263/477)<br>(50.6, 59.5)	55.7<br>(263/472)<br>(51.2, 60.1)	30.1<br>(92/306)<br>(27.1, 32.9)	32.1<br>(99/308)<br>(29.1, 35.1)	8.7<br>(25/288)<br>(6.2, 11.8)	9.9<br>(29/292)<br>(7.3, 13.1)	51.5<br>(306/594)<br>(47.6, 55.4)	3.33<br>(306/92)<br>(3.04, 3.69)
	HPV18+	DS	86.7<br>(13/15)<br>(62.1, 96.3)	86.4<br>(19/22)<br>(66.7, 95.3)	50.0<br>(104/208)<br>(43.3, 56.7)	51.0<br>(103/202)<br>(44.1, 57.8)	11.1<br>(13/117)<br>(8.1, 13.2)	16.1<br>(19/118)<br>(12.6, 18.8)	1.9<br>(2/106)<br>(0.5, 5.2)	2.8<br>(3/106)<br>(1.0, 6.7)	52.5<br>(117/223)<br>(46.0, 58.9)	9.00<br>(117/13)<br>(7.59, 12.38)
		Cytology	80.0<br>(12/15)<br>(54.8, 93.0)	77.3<br>(17/22)<br>(56.6, 89.9)	60.1<br>(125/208)<br>(53.3, 66.5)	60.9<br>(123/202)<br>(54.0, 67.4)	12.6<br>(12/95)<br>(8.8, 15.6)	17.7<br>(17/96)<br>(13.2, 21.6)	2.3<br>(3/128)<br>(0.8, 5.2)	3.9<br>(5/128)<br>(1.8, 7.3)	42.6<br>(95/223)<br>(36.2, 49.0)	7.92<br>(95/12)<br>(6.40, 11.37)
	12 Other HPV+	DS	86.2<br>(81/94)<br>(77.8, 91.7)	86.8<br>(99/114)<br>(79.4, 91.9)	55.6<br>(1563/2812)<br>(53.7, 57.4)	55.9<br>(1581/2827)<br>(54.1, 57.7)	6.1<br>(81/1330)<br>(5.5, 6.5)	7.4<br>(99/1345)<br>(6.7, 7.9)	0.8<br>(13/1576)<br>(0.5, 1.3)	0.9<br>(15/1596)<br>(0.6, 1.5)	45.8<br>(1330/2906)<br>(44.0, 47.6)	16.42<br>(1330/81)<br>(15.27, 18.20)
		Cytology	67.0<br>(63/94)<br>(57.0, 75.7)	65.8<br>(75/114)<br>(56.7, 73.9)	64.8<br>(1823/2812)<br>(63.0, 66.6)	65.0<br>(1837/2827)<br>(63.2, 66.7)	6.0<br>(63/1052)<br>(5.1, 6.8)	7.0<br>(75/1065)<br>(6.1, 7.9)	1.7<br>(31/1854)<br>(1.2, 2.2)	2.1<br>(39/1876)<br>(1.6, 2.6)	36.2<br>(1052/2906)<br>(34.5, 37.9)	16.70<br>(1052/63)<br>(14.74, 19.58)

CI, confidence intervals; CIN, cervical intraepithelial neoplasia; DS, Dual-stain; PPV, positive predictive value; NPV, negative predictive value

Supplementary Table 6. Triage performance of Dual-stain and cytology, alone or in combination with HPV16/18 genotyping for detecting ≥CIN2 and ≥CIN3 in cobas 4800 HPV-positive women: baseline and cumulative 1-year data

		**Sensitivity % (n/N) 95%CI**		**Specificity % (n/N) 95%CI**		**PPV % (n/N) 95%CI**		**1-NPV % (n/N) 95%CI**		**Positivity rate % (n/N) 95%CI**	**Number of baseline colpos per disease detected % (n/N) 95%CI**
**Disease status**	**Triage with**	**Baseline**	**Cumulative**	**Baseline**	**Cumulative**	**Baseline**	**Cumulative**	**Baseline**	**Cumulative**	**Baseline**	**Baseline**
**≥CIN2**	DS	86.5 (460/532) (83.3, 89.1)	84.7 (530/626) (81.6, 87.3)	56.5 (1804/3191) (54.8, 58.2)	57.5 (1804/3139) (55.7, 59.2)	24.9 (460/1847) (23.9, 25.9)	28.4 (530/1865) (27.3, 29.5)	3.8 (72/1876) (3.1, 4.7)	5.1 (96/1900) (4.2, 6.0)	49.6 (1847/3723) (48.1, 51.1)	4.02 (1847/460) (3.87, 4.18)
	Cytology	66.0 (351/532) (61.8, 69.9)	64.4 (403/626) (60.5, 68.0)	65.5 (2089/3191) (63.8, 67.1)	66.0 (2073/3139) (64.4, 67.7)	24.2 (351/1453) (22.7, 25.6)	27.4 (403/1469) (25.9, 28.9)	8.0 (181/2270) (7.1, 8.9)	9.7 (223/2296) (8.8, 10.7)	39.0 (1453/3723) (37.5, 40.6)	4.14 (1453/351) (3.91, 4.40)
	HPV16/18 GT + DS	89.5 (476/532) (86.6, 91.8)	88.2 (552/626) (85.4, 90.5)	47.6 (1520/3191) (45.9, 49.4)	48.5 (1522/3139) (46.7, 50.2)	22.2 (476/2147) (21.4, 22.9)	25.4 (552/2169) (24.6, 26.3)	3.6 (56/1576) (2.8, 4.5)	4.6 (74/1596) (3.8, 5.7)	57.7 (2147/3723) (56.1, 59.2)	4.51 (2147/476) (4.36, 4.67)
	HPV16/18 GT + cytology	75.9 (404/532) (72.1, 79.4)	74.8 (468/626) (71.2, 78.0)	54.1 (1726/3191) (52.4, 55.8)	54.7 (1718/3139) (53.0, 56.5)	21.6 (404/1869) (20.6, 22.6)	24.8 (468/1889) (23.7, 25.9)	6.9 (128/1854) (6.0, 7.9)	8.4 (158/1876) (7.4, 9.5)	50.2 (1869/3723) (48.6, 51.8)	4.63 (1869/404) (4.42, 4.86)
**≥CIN3**	DS	90.3 (204/226) (85.7, 93.5)	90.2 (238/264) (86.0, 93.2)	53.0 (1854/3497) (51.4, 54.7)	53.5 (1874/3501) (51.9, 55.2)	11.0 (204/1847) (10.5, 11.6)	12.8 (238/1865) (12.1, 13.3)	1.2 (22/1876) (0.8, 1.7)	1.4 (26/1900) (0.9, 1.9)	49.6 (1847/3723) (48.0, 51.2)	9.05 (1847/204) (8.65, 9.56)
	Cytology	73.9 (167/226) (67.8, 79.2)	72.3 (191/264) (66.7, 77.4)	63.2 (2211/3497) (61.6, 64.8)	63.5 (2223/3501) (61.9, 65.1)	11.5 (167/1453) (10.6, 12.4)	13.0 (191/1469) (12.0, 14.0)	2.6 (59/2270) (2.1, 3.2)	3.2 (73/2296) (2.6, 3.8)	39.0 (1453/3723) (37.5, 40.6)	8.70 (1453/167) (8.09, 9.47)
	HPV16/18 GT + DS	94.2 (213/226) (90.4, 96.6)	94.3 (249/264) (90.8, 96.5)	44.7 (1563/3497) (43.1, 46.3)	45.2 (1581/3501) (43.5, 46.8)	9.9 (213/2147) (9.5, 10.3)	11.5 (249/2169) (11.0, 11.9)	0.8 (13/1576) (0.5, 1.4)	0.9 (15/1596) (0.6, 1.5)	57.7 (2147/3723) (56.1, 59.2)	10.08 (2147/213) (9.72, 10.54)
	HPV16/18 GT + cytology	86.3 (195/226) (81.2, 90.2)	85.2 (225/264) (80.4, 89.0)	52.1 (1823/3497) (50.5, 53.8)	52.5 (1837/3501) (50.8, 54.1)	10.4 (195/1869) (9.8, 11.0)	11.9 (225/1889) (11.2, 12.5)	1.7 (31/1854) (1.2, 2.3)	2.1 (39/1876) (1.6, 2.7)	50.2 (1869/3723) (48.6, 51.8)	9.58 (1869/195) (9.11, 10.20)

CI, confidence intervals; CIN, cervical intraepithelial neoplasia; DS, Dual-stain; GT, genotypes; PPV, positive predictive value; NPV, negative predictive value

Supplementary Figure 1. Flow of subjects through baseline and follow-up phases of the IMPACT trial

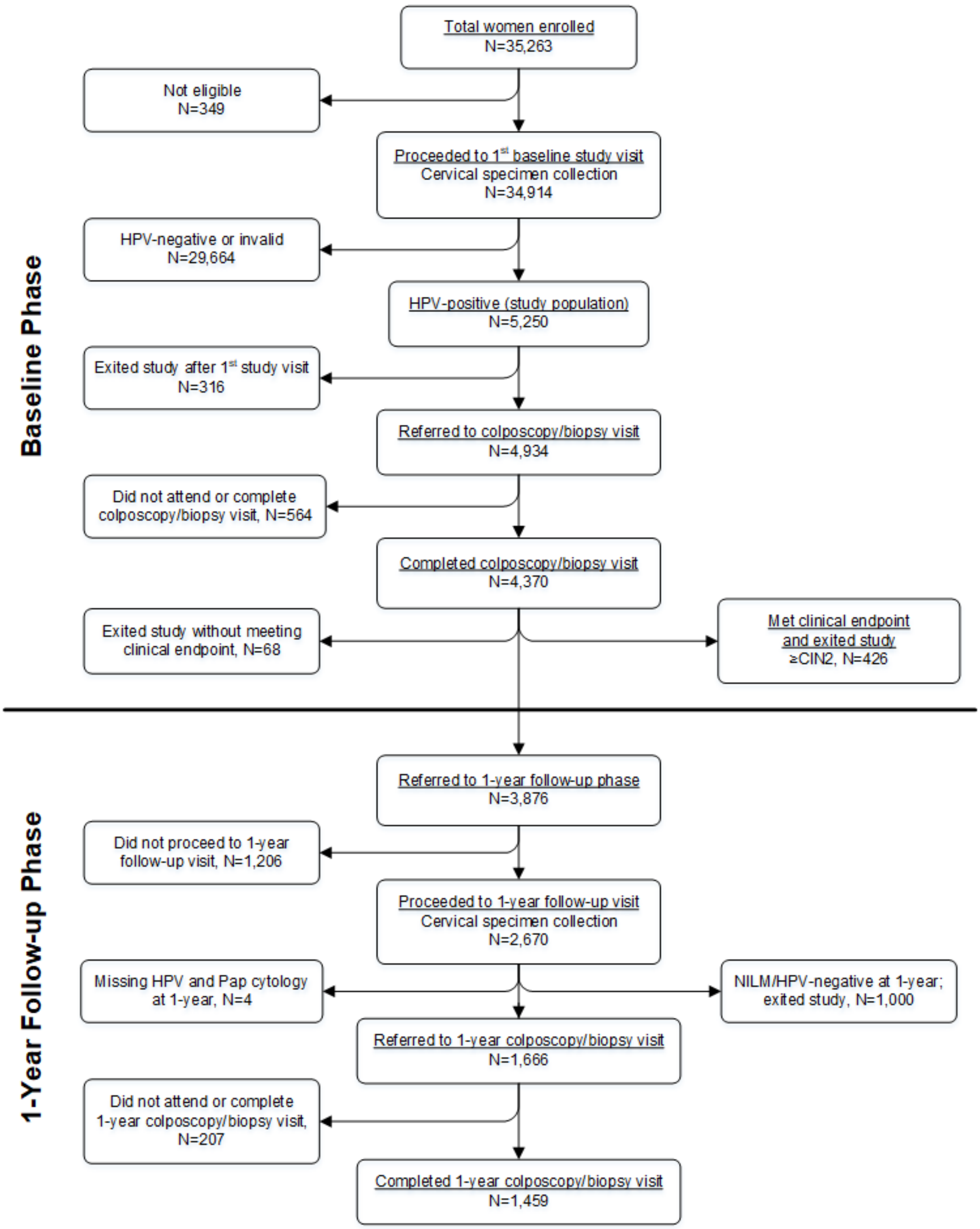

Supplementary Figure 2. CONSORT diagram. Triage of cobas 4800 HPV-positive women

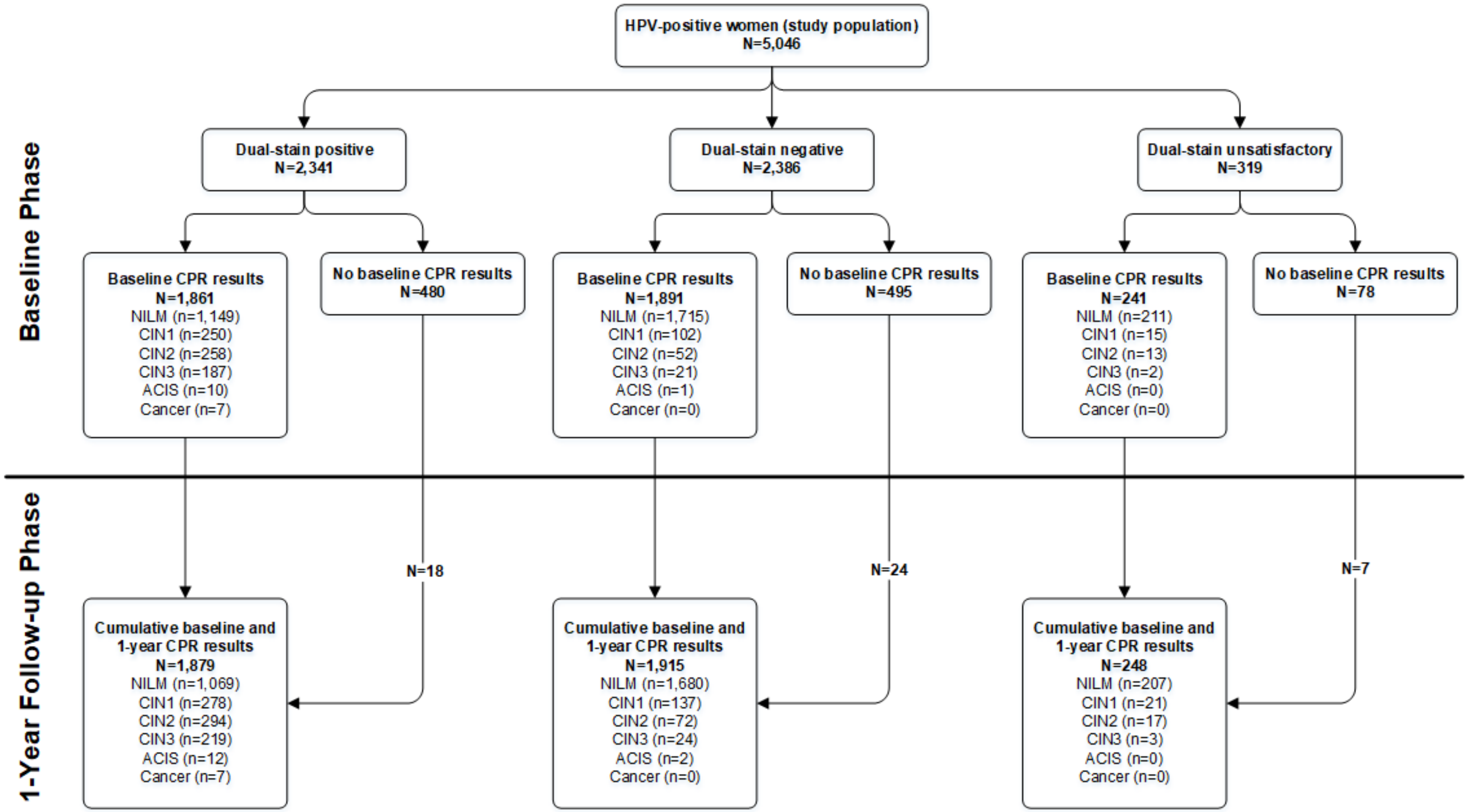

Note: There were 480 (DS positive), 495 (DS negative), and 78 (DS unsatisfactory) cases with no baseline CPR results, which made them eligible to be referred to the 1-year follow-up phase. Among them, there were 18, 24, and 7 cases in the DS positive, DS negative, and DS unsatisfactory categories, respectively, which had valid 1-year CPR results. CPR, central pathology review; NILM, negative for intraepithelial lesion or malignancy; CIN, cervical intraepithelial neoplasia; ACIS, adenocarcinoma in-situ

Supplementary Figure 3. Risk of ≥CIN3 in cobas 4800 HPV-positive women dependent on HPV genotype group, cytology, and Dual-stain results

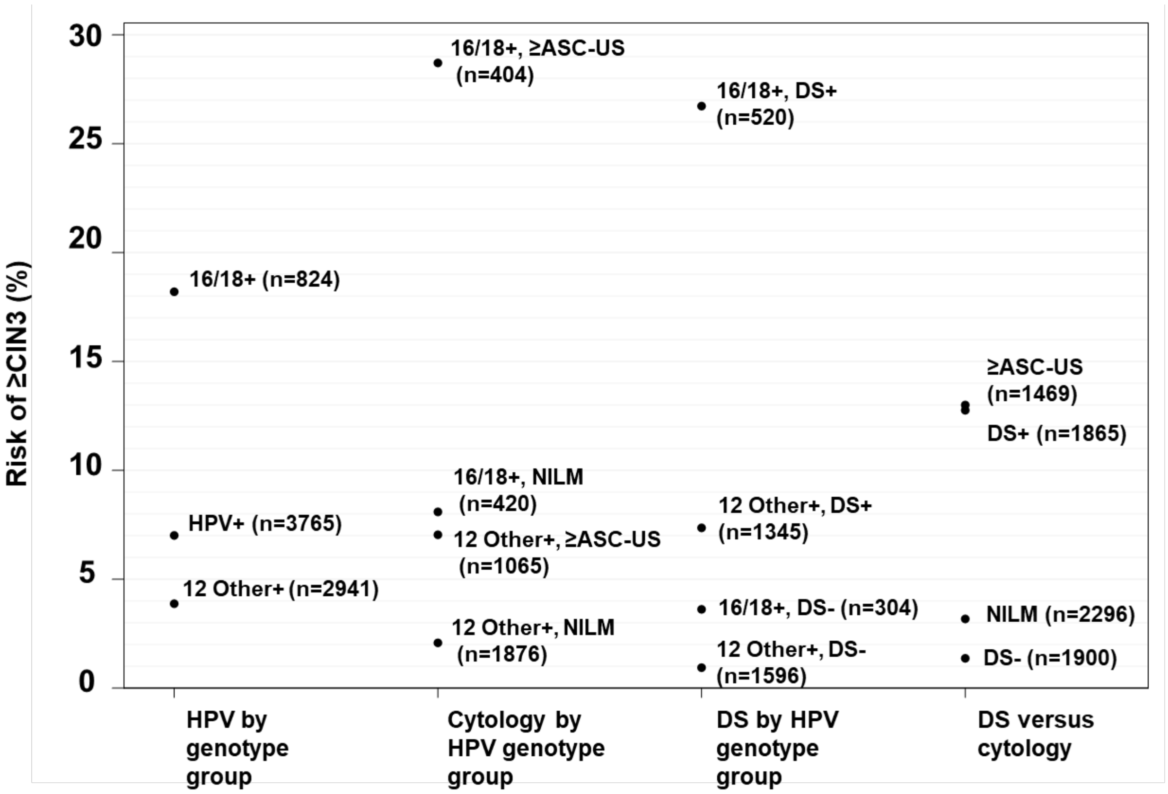

Cumulative 1-year risks of ≥CIN3 for HPV genotype groups (16/18+; 12 Other+), cytology (NILM; ≥ASC-US), and Dual-stain (DS+; DS-) test results are plotted on the y-axis, with the number of subjects within the respective categories indicated
